# Glucose-Lowering and the Risk of Cardiovascular Events With Antidiabetic Therapies: A Systematic Review and Additive-Effects Network Meta-Analysis

**DOI:** 10.3389/fcvm.2022.876795

**Published:** 2022-04-29

**Authors:** Luiz Sergio Fernandes de Carvalho, Ana Claudia Cavalcante Nogueira, Isabella Bonilha, Beatriz Luchiari, Alexander Benchimol, Carlos Eduardo Barra Couri, Jairo Lins Borges, Joaquim Barreto, Andrei C. Sposito

**Affiliations:** ^1^Laboratory of Data for Quality of Care and Outcomes Research, Clarity Healthcare Intelligence, Jundiaí, Brazil; ^2^Catholic University of Brasília (UCB), Brasilia, Brazil; ^3^Atherosclerosis and Vascular Biology Laboratory (Atherolab), Cardiology Division, University of Campinas (Unicamp), Campinas, Brazil; ^4^Cardiology Department, State Institute of Diabetes and Endocrinology, Rio de Janeiro, Brazil; ^5^Department of Hematology, University of São Paulo, São Paulo, Brazil; ^6^São Paulo Federal University (UNIFESP), São Paulo, Brazil

**Keywords:** new antidiabetic drugs, MACE, HbA1c, diabetes mellitus, meta-analysis

## Abstract

**Aim:**

To assess the impact of the HbA1c levels achieved with antidiabetic therapies (ADTs) on the risk of MACE.

**Methods:**

A systematic search was performed in PubMed, Cochrane, and ClinicalTrials. gov for RCTs published up to March 2022 reporting the occurrence of MACE and all-cause mortality in individuals with T2DM treated with all marketed ADTs, including a sample size ≥100 individuals in each study arm and follow-up ≥24 weeks. A systematic review and additive-effects network meta-analysis with random effects and a multivariate meta-regression were utilized to assess the impact of achieved HbA1c on incident MACE.

**Results:**

We included 126 RCTs with 143 treatment arms, 270,874 individuals, and 740,295 individuals-years who were randomized to an active treatment vs. control group. Among all ADTs, only therapy with SGLT2i, GLP1-RA, or pioglitazone similarly reduced the risk of MACE compared to placebo. The achievement of HbA1c ≤ 7.0% in RCTs with the 3 drug classes in the active arm was associated with an adjusted HR of 0.91 (95% CI 0.80, 0.97; *p* = 0.017) compared with HbA1c>7.0%, without affecting all-cause mortality. These results, however, were not maintained among all ADTs.

**Conclusions:**

Achieving lower glucose levels with SGLT2i, GLP1-RA, or pioglitazone is linearly associated with a reduced risk of MACEs, without affecting all-cause mortality.

**Systematic Review Registration:**

www.crd.york.ac.uk/prospero/display_record.php?ID=CRD42020213127, identifier: CRD42020213127.

## Introduction

Among individuals with type 2 diabetes mellitus (T2DM), observational studies have shown an increased risk of both macrovascular and microvascular events with increasing blood glucose levels (1, 2). Consistently, randomized clinical trials (RCTs) involving subjects with new-onset (3) or long-lasting (4–6) T2DM showed that intensive glucose control, i.e., glycated hemoglobin (HbA1c) ≤ 7.0% (53 mmol/mol), reduces the incidence of microvascular complications. Nevertheless, the RCTs that were designed to investigate the cardiovascular effects of achieving HbA1c ≤ 7.0% were not able to identify a significant decrease in the incidence of macrovascular events (4–6). Several factors may explain these negative results: (i) these trials were designed with drugs such as insulin and sulfonylureas, that share a high risk of severe hypoglycemia and a neutral or increasing effect on weight (7); (ii) as these adverse effects are positively associated with major adverse cardiovascular events (MACE) (8–10), they could attenuate any potential benefit of intensive glucose-lowering; (iii) the lower rates of MACE than originally predicted induced lower statistical power. Weight gain of ≥5% is related to an increase in blood pressure, lipid profile deterioration as well as activation of chronic inflammation and neurohumoral factors (8, 11, 12), which are also associated with the development of cardiovascular disease. In parallel, through the release of catecholamines, severe hypoglycemia may increase the risk of cardiovascular events by triggering endothelial dysfunction, decreased vasodilation and arrhythmias (13). A meta-analysis suggested that insulin and sulfonylureas could marginally attenuate the incidence of MACE by about 6%, but the statistical power was insufficient to verify this assumption (14). Therefore, it remains unclear whether the lack of evidence of cardiovascular benefit with an HbA1c ≤ 7.0% is due to the inadequacy of the statistical power or due to the side effects of some antidiabetic therapies (ADTs) such as insulin and sulfonylureas (4–6).

ADTs such as sodium-glucose cotransporter inhibitors (SGLT2i), glucagon-like peptide-1 receptor agonists (GLP1-RA), and pioglitazone, reduce MACE (15–17) via a broad spectrum of mechanisms, which may or may not be additive to their glucose-lowering effect. Moreover, these new therapies, as well as dipeptidyl peptidase-4 inhibitors (DPP4i) have a low risk of severe hypoglycemia as a common advantage compared to insulin and sulfonylureas. A large number of RCTs have been performed to prove safety with SGLT2i, DPP4i, GLP1-RA and pioglitazone, and in these studies, wide variability of HbA1c change was observed; while in some active arms, the post-treatment HbA1c was >8.0%; in others, the post-therapy HbA1c was <7.0% (18). The wide amplitude of post-treatment HbA1c levels enabled us to test the assumption that more intense glucose-lowering has an additive effect in preventing MACE when drugs not associated with severe hypoglycemia are used, *that is*, SGLT2i, DPP4i, GLP1-RA, or pioglitazone. Hence, this systematic review aimed to investigate the association between glucose-lowering in T2DM and the incidence of MACE in two sets of data: (i) all available RCTs reporting MACE to achieve sufficient statistical power, and (ii) exclusively RCTs that used ADTs associated with a low risk of severe hypoglycemia.

## Methods

### Search Strategy and Study Eligibility

This systematic review was carried out in accordance with the guidelines of the Preferred Reporting Items for Systematic Reviews and Meta-analyses for Network Meta-analysis (PRISMA-NMA) (19). A detailed description of all the procedures is provided in the Supplementary Material. Briefly, the review was performed by searching keywords in the following databases: Medline (PubMed), ClinicalTrials.gov Cochrane Central Register of Controlled Trials, Embase, European Union Clinical Trials Register, and World Health Organization (WHO) International Clinical Trials Registry Platform electronic database entries until March 2022. Briefly, studies were included in the meta-analysis if they met all of the following criteria: (1) included individuals with diabetes mellitus and ADTs; (2) randomized double-blinded controlled study design; (3) sample size ≥100 individuals in each study arm; (4) follow-up ≥ 24 weeks; and (5) report of 3-point major adverse cardiovascular events (MACE) in both control and intervention groups. We excluded phase 1 or 2 RCTs, studies in type 1 diabetes, and studies without adequate information on outcomes or without a control group. The meta-analysis was registered in the International Prospective Register of Systematic Reviews (CRD42020200649) and detailed search terms, data sources are available in Supplementary Material.

### Data Source and Search

Queries of literature were performed using the electronic databases Medline (PubMed), ClinicalTrials.gov, Cochrane Central Register of Controlled Trials, Embase, European Union Clinical Trials Register and World Health Organization (WHO) International Clinical Trials Registry Platform. The search included all submitted articles until 21^st^ March 2022 with no restriction to submission date or language, however all articles relevant to this study were published in English. The search was filtered to include only randomized controlled trials (RCTs) involving humans. Systematic reviews and meta-analyses were also evaluated to identify other relevant RCTs that were eventually missing by using the search terms. The search terms used are described in Table 1.

**Table 1:** Search terms used in systematic review protocol.

**Items**	**N**.	**Terms**
Disease	#1	“type 2 diabetes mellitus”[MeSH Terms] OR (“type 2 diabetes”[All Fields]
SGLT2i	#2	“canagliflozin”[MeSH Terms] OR “canagliflozin”[All Fields]
	#3	“empagliflozin”[MeSH Terms] OR “empagliflozin”[All Fields]
	#4	“dapagliflozin”[MeSH Terms] OR “dapagliflozin”[All Fields]
	#5	“ertugliflozin”[MeSH Terms] OR “ertugliflozin”[All Fields]
	#6	“ipragliflozin”[MeSH Terms] OR “ipragliflozin”[All Fields]
	#7	“tofogliflozin”[MeSH Terms] OR “tofogliflozin”[All Fields]
GLP-1A	#8	”lixisenatide“[MeSH Terms] OR “lixisenatide”[All Fields]
	#9	“liraglutide”[MeSH Terms] OR “liraglutide”[All Fields]
	#10	“semaglutide”[MeSH Terms] OR “semaglutide”[All Fields]
	#11	“albiglutide”[All Fields] OR “rGLP-1 protein”[Supplementary Concept] OR “rGLP-1 protein”[All Fields]
	#12	“exenatide”[MeSH Terms] OR “exenatide”[All Fields]
	#13	“dulaglutide”[Supplementary Concept] OR “dulaglutide”[All Fields]
DPP4i	#14	“alogliptin”[MeSH Terms] OR ”alogliptin”[All Fields]
	#15	“linagliptin”[MeSH Terms] OR ”linagliptin”[All Fields]
	#16	“omarigliptin”[MeSH Terms] OR ”omarigliptin”[All Fields]
	#17	“saxagliptin”[MeSH Terms] OR ”saxagliptin”[All Fields]
	#18	“sitagliptin”[MeSH Terms] OR ”sitagliptin”[All Fields]
	#19	“vildagliptin”[MeSH Terms] OR ”vildagliptin”[All Fields]
Sulfonylurea	#20	”glibenclamide“[MeSH Terms] OR “glibenclamide”[All Fields]
	#21	”gliclazide“[MeSH Terms] OR “gliclazide”[All Fields]
	#22	“glimepiride”[MeSH Terms] OR “glimepiride”[All Fields]
	#23	“glipizide”[MeSH Terms] OR “glipizide”[All Fields]
Thiazolidinedione	#24	“pioglitazone”[MeSH Terms] OR “pioglitazone”[All Fields]
Study design	#25	“randomized controlled trials”[MeSH Terms] OR “randomized controlled trial”[All Fields] OR controlled clinical trial[Publication Type])
Filters	#26	“humans”[MeSH Terms]
**Strings**
1^st^ search - SGLT2i	#27	#1 AND (#2 OR #3 OR #4 OR #5 OR #6 OR #7) AND #25 AND #26
2^nd^ search - GLP1-RA	#28	#1 AND (#8 OR #9 OR #10 OR #11 OR #12 OR #13) AND #25 AND #26
3^th^ search - DPP4i	#29	#1 AND (#14 OR #15 OR #16 OR #17 OR #18 OR #19) AND #25 AND #26
4^th^ search - Sulfonylurea	#30	#1 AND (#20 OR #21 OR #22 OR #23) AND #25 AND #26
5^th^ search - Thiazolidinedione	#31	#1 AND (#24) AND #25 AND #26

### Data Extraction and Quality Assessment

Data were extracted by four authors (A.C.C.N., R.M.R.C., I.B., and B.L.), and any inconsistencies were resolved after discussion with the senior researchers (A.C.S. and L.S.F.C.). Extracted data included the name of first author, year of publication, sample size, duration of follow-up, patient characteristics (sex, age, race), duration of diabetes, active (or experimental) and comparative drug, history of cardiovascular events and heart failure, average systolic and diastolic blood pressure, weight, body mass index (BMI), glycated hemoglobin values (HbA1c), estimated glomerular filtration rate (eGFR), clinical outcomes, and adverse events.

For the meta-analyses, the 126 RCTs provided data of 270,874 patients randomized to an active treatment (DPP4i [32 trials] or DPP4i + TZD [3 trials] or GLP1-RA [44 trials] or GLP1-RA + Insulin [5 trials] or GLP1-RA + TZD [1 trial] or SGLT2i [37 trials] or SGLT2i + DPP4i [4 trials] or SGLT2i + GLP1-RA [2 trials] or Sulfonylurea [2 trials] or Sulfonylurea + Insulin [3 trials] or TZD [10 trials] vs. control (placebo [63 trials] or DPP4i [14 trial] or DPP4i + Metformin [1 trial] or DPP4i + TZD [1 trial] or Insulin [6 trials] or Metformin [7 trials] or Sulfonylurea [28 trials] or Sulfonylurea + Insulin [3 trial] or TZD [7 trials] or GLP1-RA [1 trial] or SGLT2i [8 trials]).

### Data Synthesis and Statistical Analyses

The primary endpoint was defined as 3-point MACE according to the definition of the study, representing a combination of nonfatal myocardial infarction, nonfatal stroke, and cardiovascular death. Secondary endpoints were defined as (i) non-fatal myocardial infarction and (ii) all-cause death. As tertiary endpoints we also studied the relationship between (i) weight change or (ii) incidence of severe hypoglycemia and the occurrence of MACE. We used an additive component network meta-analysis (CNMA) framework to perform an indirect comparison between the drugs. This model is based on the premise that the effect of a treatment combination is the sum of the effects of its components, which implies that the common components cancel each other out in comparisons. An additive CNMA model can be used to evaluate the influence of individual components and their combinations, in contrast to standard network meta-analyses (NMA), which considers that all existing (single or combined) treatments are different nodes in the network. The advantage of employing CNMA here is to identify potential sources of bias related to drug combinations.

Dichotomous variables are reported as percentages, while continuous variables are reported as mean±SD or median (interquartile range). To identify the potential effects of therapies on clinical outcomes, we calculated the hazard ratios (HRs) with random-effects CNMA. We assessed statistical heterogeneity between trials using the I^2^ statistic (with 95% CIs), which is derived from Cochran's Q [100 × (Q–df÷Q)] and provides a measure of the proportion of overall variation attributable to between-trial heterogeneity. We investigated potential sources of heterogeneity between the RCTs through meta-regression analyses using the restricted maximum-likelihood estimator and the method by Knapp and Hartung (20) for adjusting test statistics and confidence intervals.

We performed prespecified multivariate meta-regressions to address the anticipated heterogeneity among RCTs in meta-regressions and to address the imbalance between RCTs that achieved HbA1c <7.0% vs. HbA1c>7.0% at the study end in the active arm. The adjusting variables were defined after data collection and were chosen if imbalance was identified between achieved HbA1c subgroups, namely: (i) the time since the diagnosis of T2DM, (ii) type of ADT in the active arm, and (iii) the length of follow-up time. To test for publication bias, we created funnel charts and performed the Egger test.

Sensitivity analyses were carried out to evaluate the stability of meta-regression models after: (i) to address whether largest trials are critical to results presented as main analyses, we sequentially excluded trials with the largest exposures (sample size ^*^ follow-up time); (ii) exclusion of the ACCORD, VADT, and ADVANCE trials (4–6), RCTs that did not compare drug classes, but specific HbA1c targets. To estimate the effect of the treatment, a two-tailed *p* <0.05, was considered statistically significant. *Post-hoc* statistical power estimation was carried out using the method described by Jackson et al. (21). The extracted data were analyzed using R v4.0.1 (2020, Auckland, New Zealand) and *discomb, metaviz*, and *metafor* packages.

## Results

**Figure 1:**
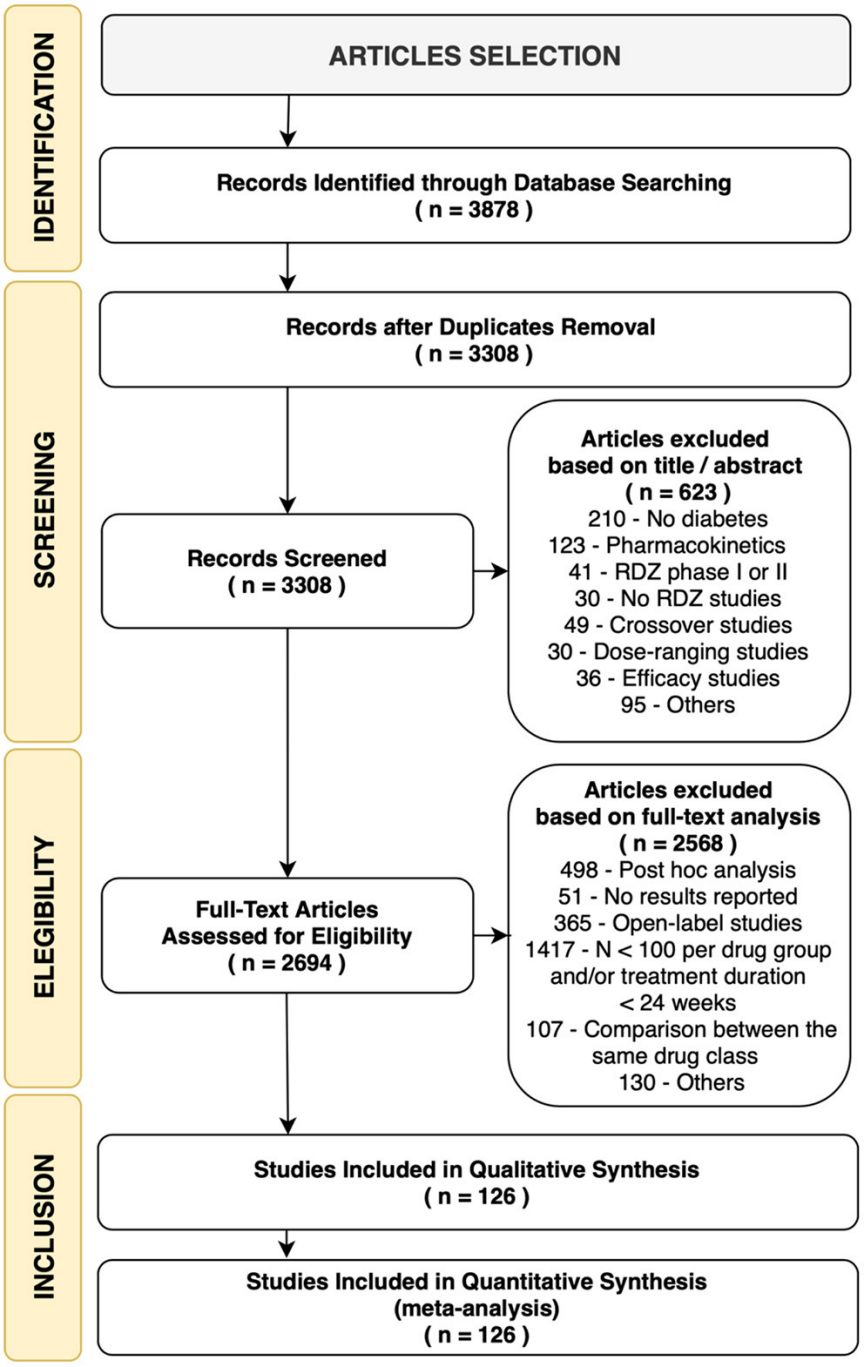
Flow diagram of the trials' selection process. RDZ, randomization.

**Figure 2:**
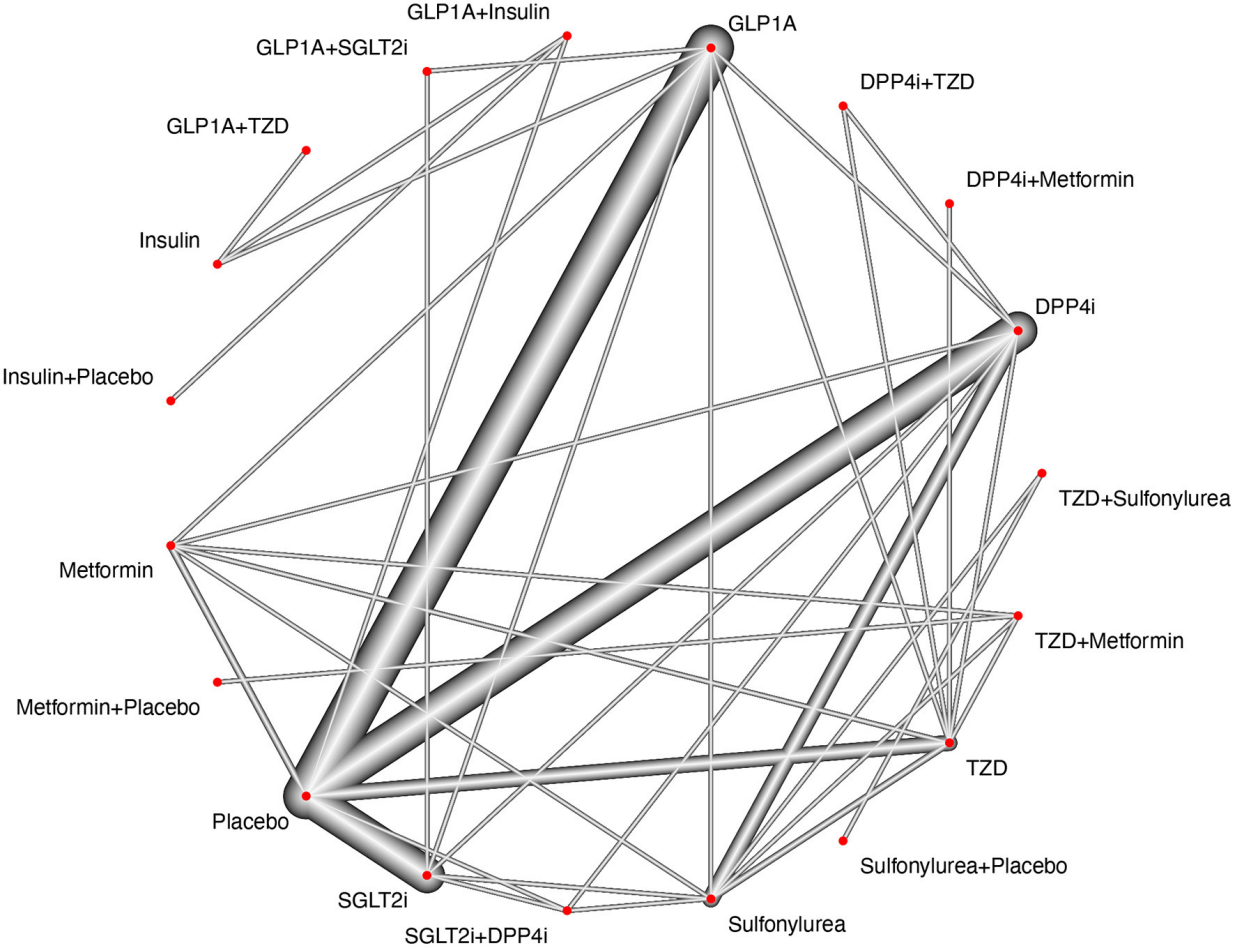
Study network. The study network presents sharp connections between placebo vs. GLP1-RA, placebo vs. SGLT2i, placebo vs. DPP4i and placebo vs. TZD (pioglitazone). The nodes for Sulfonylurea, TZD, DPP4i, GLP1-RA, SGLT2i are also tightly connected with most treatment arms, however, it is possible to observe a poor connection between drug combinations (SGLT2i+GLP1-RA, GLP1-RA1+Insulin, SGLT2i+DPP4i, GLP1-RA+TZD, etc) and other treatment arms. By using additive component network meta-analysis, it is possible to account for both single treatments and combinations, but the combinations may be more prone to depend on the effect of indirect comparisons if paralleled to single treatment comparisons.

We identified 3,878 citations using the search terms and platforms mentioned above. After excluding duplicates, 3,308 articles remained. We further excluded 623 articles that were unsuitable according to the title and abstract. An additional 2,572 studies were excluded after full-text evaluation, with no results reported, open-label studies, *post-hoc* analyses, comparisons of the same drug classes, n <100 per group, and/or treatment duration <24 weeks (details on Supplementary Data and Figure 1). We ended this extraction with 228 trials to be included for qualitative synthesis and meta-analysis, but among them, only 126 RCTs reported MACE. The flow diagram of the selection process and the study network are shown in Figures 1, 2, respectively.

These 126 RCTs provided data of 270,874 patients within 143 active arms, mean 1.48 ± 1.12 years of follow-up and total of 740,295 patient-years that were randomized to an active treatment vs. control (full description of study arms is available in Supplementary Material). The baseline characteristics of the enrolled individuals within the trials are shown in Supplementary Table S1. All studies presented a low risk of bias as assessed by the Cochrane Collaboration tool for assessing the risk of bias (22) (see Supplementary Table S2) and were deemed high quality by the GRADE system (23), except for two RCTs: the CONFIDENCE trial and Abdul-ghani (2017). As shown in Supplementary Table S3 and Supplementary Figure S1, there was no significant publication bias in the funnel plots, and there was no significant small study bias in the Egger tests.

The mean age was 57.3 ± 9.6 years (45% female). Individuals had the diagnosis of diabetes for 7.5 ± 5.7 years, 82% were on metformin at baseline, 21% were on insulin, 51.2% were enrolled in trials with individuals with prior MI or stroke at baseline and 15% of the enrolled individuals had heart failure at baseline. The mean baseline body mass index (BMI) across trials was 31.0 ± 5.2 kg/m^2^, body weight was 86.5 ± 18.7 kg, systolic blood pressure (SBP) was 130.8 ± 6 mmHg, diastolic blood pressure (DBP) was 78.2 ± 5 mmHg, estimated glomerular filtration rate was 80.3 ml/min/1.73 m^2^ and HbA1c 8.0 ± 0.9%.

### Primary Outcome

**Figure 3:**
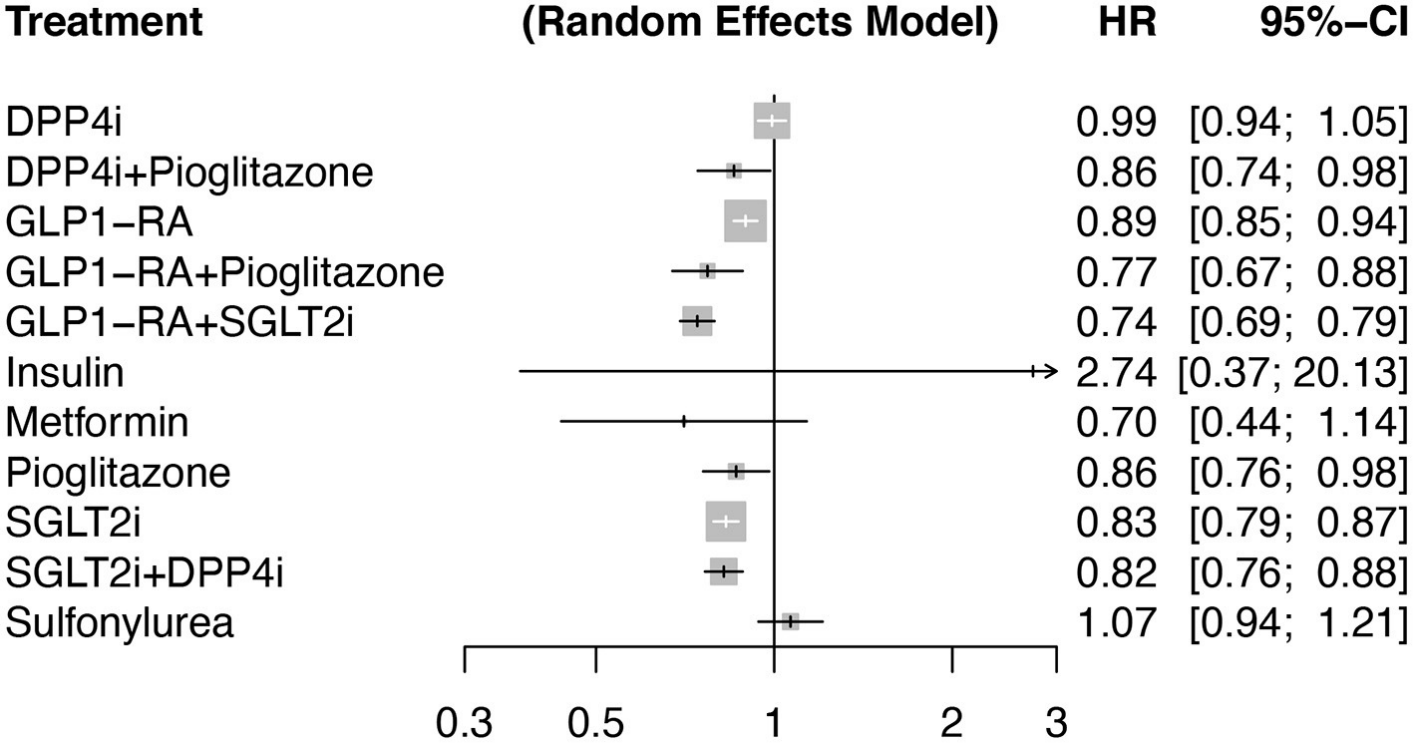
Forest plot comparing antidiabetic therapies for the occurrence of major cardiovascular adverse events (MACE) in an additive effects network meta-analysis with a random-effects model. The reference group was considered as placebo. Number of pairwise comparisons was 140 (140 studies with >100 individuals per arm), number of treatments (*n* = 12), number of designs (*n* = 25) with 7 active components. Heterogeneity / inconsistency analysis showed tau2 = 0; tau = 0; I^2^ = 0% [0.0; 6.5%]; Q = 111.72 (*p* = 0.91). Among TZDs (thiazolidinediones) only pioglitazone was included.

The primary outcome (MACE) occurred in 10,354 individuals assigned to active treatment (median across trials of 48.7/1,000 patient-years [interquartile range (IQR) 19.1]) and 10,370 individuals assigned to the control group (median of 55.0/1,000 patient-years [IQR 20.3]). In an additive model network meta-analysis with random effects, DPP4i alone (*p* = 0.76), insulin (*p* = 0.32), sulfonylurea alone (*p* = 0.32), and metformin alone (*p* = 0.15) showed a neutral effect on the risk of MACE compared to placebo with no heterogeneity (Q = 111; I^2^ = 0%, *p* = 0.91). Contrarily, SGLT2i alone, GLP1-RA alone, and pioglitazone alone reduced the risk of MACE compared to placebo with an HR of 0.83 [95%CI 0.79, 0.87, *p* <0.001, 0.89 [95% CI 0.85, 0.94, *p* <0.0001] and 0.86 [95% CI 0.76, 0.98, *p* = 0.024], respectively) (Figure 3). The meta-analysis with and without additive effects yielded similar results (p for difference 0.54).

### Secondary Outcomes

To evaluate the specificity of the findings, as secondary outcomes, we evaluated the relationship between HbA1c change and levels and secondary outcomes: (i) the HR for all-cause deaths and (ii) the HR for non-fatal myocardial infarction. In meta-regression analyses, no relationship was found between the two secondary outcomes and achieved HbA1c at the study end (Supplementary Figures S3A,B), nor with the differential change in HbA1c between the active and control arms. Again, both the meta-analysis with and without additive effects showed similar effect (p for difference 0.91 for all-cause death and 0.56 for non-fatal myocardial infarction).

### Tertiary Outcomes

We also evaluated the relationship between weight change and the HR for MACE in order to confirm this assumption (Supplementary Figure S4). Among studies reporting neutral weight change in the active treatment arm (0 to 1kg loss, 20 studies) and those reporting weight gain (>0 kg, 26 studies) there was no evidence of reduction in MACE with HR of 0.96 (95% CI 0.78 to 1.11, *p* = 0.32) and 0.94 (95% CI 0.82 to 1.07, *p* = 0.23), respectively. Among studies reporting weight loss superior to 1 kg in the active treatment arm, the HR of 0.82 (95% CI 0.77 to 0.87, *p* <0.001).

The incidence of severe hypoglycemia was evaluated among the 91 studies that had this information available. With 5 active components (DPP4i, GLP1-RA, pioglitazone, SGLT2i and sulfonylureas), only sulfonylureas were associated with severe hypoglycemia with an HR of 6.72 (95% CI 4.4485; 10.1540, *p* <0.0001), while SGLT2i reduced the risk of severe hypoglycemia with HR of 0.6870 (95% CI 0.5584; 0.8452, *p* = 0.0004) and DPP4i, GLP1-RA, pioglitazone were neutral. Of note, there is high heterogeneity regarding this analysis (I^2^ = 36.3% (95% CI 17.0%; 51.0%, p for heterogeneity = 0.0006), indicating the relevance of hypoglycemia as a major covariable for these analyses. Indeed, as seen in Supplementary Figure S5, among RCTs where severe hypoglycemia is more frequent in the control arm than in the active arm (mean of 8.1% incidence in the control vs. 6.0% incidence in the active arm) there is a clear relationship between study drug and reduced risk of MACE (*n* = 138,073). However, among studies reporting severe hypoglycemia more frequently in the active arm than in the control arm (mean of 9.3% incidence in the active vs. 8.5% incidence in the control arm) we saw a neutral relationship between study drug and the risk of MACE (*n* = 77,395).

### Meta-Regression Analyses

**Figure 4:**
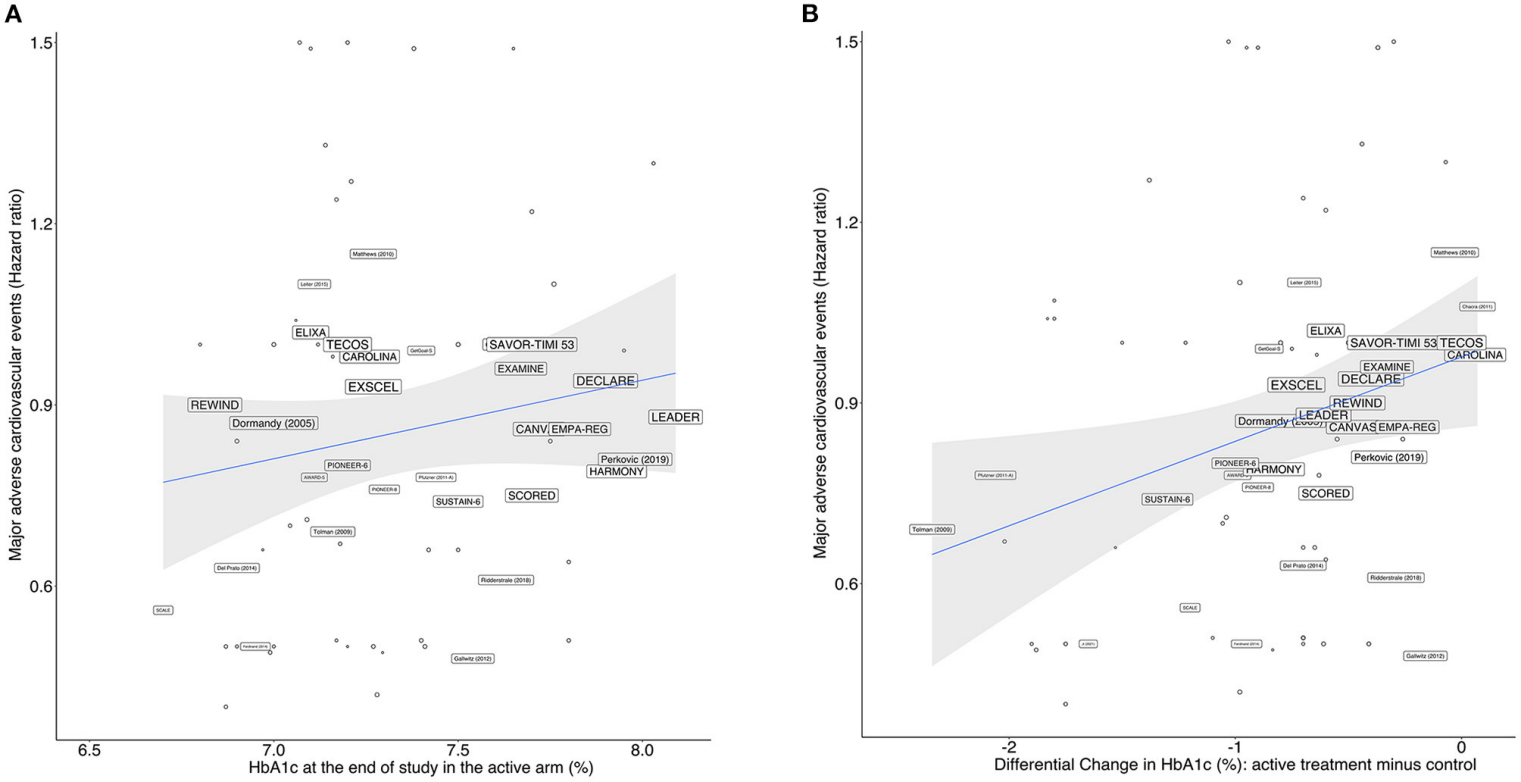
Meta-regression model for all drugs showing the relationship between the HR for MACE vs.: **(A)** achieved HbA1c levels at study end in the active arms of each trial, and **(B)** differential change in HbA1c levels between active and control arms. The size of the trials' name corresponds to their proportional weights in the regression.

As predicted during the study design, we observed that the RCTs contrasted largely in terms of glucose-lowering efficacy, with absolute reductions in HbA1c varying between −2.2% and 0 in the active arms compared to their respective control arms. We performed a prespecified bivariate meta-regression analysis with all 143 study arms, including DPP4i, pioglitazone, GLP1-RA, insulin, metformin, sulfonylurea, and SGLT2i in the active arms. RCTs reporting post-treatment HbA1c ≤ 7.0% were not associated (p for difference=0.25; I^2^ = 5.31%) with the risk of MACE compared to those with HbA1c >7.0% (Table 2). As a continuous variable, each 1% decrease in HbA1c was also not associated (p for difference=0.10; I^2^ = 12.12%) with the incidence of MACE (Table 2 and Figure 4B). The achieved (*post-hoc*) statistical power (1 – β) for comparing post-treatment HbA1c ≤ 7.0% vs >7.0% was 89%, considering the random-effect model, a two-tailed test, and summary effect size of 0.0124.

**Table 2:** Bivariate and multivariate meta-regression models with the hazard ratio for major adverse cardiovascular events (MACE) as dependent variable.

	**HR**	**95% CI**		***p***
		**Lower bound**	**Upper bound**	
**Bivariate analyses**				
Achieved HbA1c at study end in the active arm (HbA1c ≤ 7.0% vs HbA1c > 7.0%) vs MACE
All trials^‡^ (143 study arms; *n* = 270,874; 20,724 events)	0.9579	0.8117	1.1097	0.256
SGLT2i, DPP4i, Pioglitazone or GLP1-RA in the active arm (73 study arms^*^; *n* = 197,498; 17,073 events)	0.9062	0.7985	0.9673	0.017
Change in HbA1c in the active arm compared to control (each reduction of 1%) vs MACE
All trials^∅^ (143 study arms; n=270,874; 20,724 events)	0.8979	0.7249	1.0171	0.109
SGLT2i, DPP4i, Pioglitazone or GLP1-RA in the active arm^◇^ (73 study arms^*^; *n* = 197,498; 17,073 events)	0.8477	0.7395	0.9246	<0.001
				
Multivariate analyses^**^			
Achieved HbA1c at study end in the active arm (HbA1c ≤ 7.0% vs. HbA1c > 7.0%) vs. MACE
All trials^‡‡^ (121 study arms^§^; *n* = 243,713; 18,029 events)	0.8785	0.6471	1.0820	0.162
SGLT2i, DPP4i, Pioglitazone or GLP1-RA in the active arm (60 study arms^*^^§^; *n* = 191,032; 16,126 events)	0.8252	0.7111	0.9512	0.009
Change in HbA1c in the active arm compared to control (each reduction of 1%) vs. MACE
All trials^øø^ (119 study arms^§^; *n* = 240,227; 17,648 events)	0.8837	0.7019	0.9991	0.049
SGLT2i, DPP4i, Pioglitazone or GLP1-RA in the active arm^◇◇^ (60 study arms^*^^§^; *n* = 191,032; 16,126 events)	0.9046	0.7513	0.9798	0.011

* *Excluding RCTs with outlier HRs for MACE, defined as HR ≥ 2.0 or HR ≤ 0.5*.

** *Adjusted for Time since the diagnosis of T2DM, follow-up time and type of ADT in the active arm. Adjusting variables were selected for their association with HR for MACE in bivariate analyses*.

§ *Some RCTs had missing data for covariates*.

‡ *I^2^: 5.31%, QE = 83.17 (p = 0.95); tau2: 0.010 (SE = 0.022)*.

*I^2^: 12.12%, QE = 53.32 (p = 0.93); tau2: 0.010 (SE = 0.022)*.

ø *I^2^: 2.76%, QE = 92.10 (p = 0.98); tau2: 0.010 (SE = 0.022)*.

◇ *I^2^: 6.1%, QE = 67.55 (p = 0.98); tau2: 0.008 (SE = 0.023)*.

‡‡ *I^2^: 0%, QE = 73.21 (p = 0.95); tau2: 0.014 (SE = 0.012)*.

* I^2^: 0%, QE = 7.29 (p = 0.20); tau2: 0.010 (SE = 0.021)*.

øø *I^2^: 0%, QE = 85.56 (p = 0.98); tau2: 0.018 (SE = 0.027)*.

◇◇ *I^2^: 0%, QE = 77.32 (p = 0.96); tau2: 0.008 (SE = 0.028)*.

A second analysis was performed including only RCTs with SGLT2i, DPP4i, pioglitazone, or GLP1-RA in the active arm (Table 2). In this subgroup of RCTs, we found associations between MACE incidence and both HbA1c values achieved after therapy and the absolute change in HbA1c; post-treatment HbA1c ≤ 7.0% was associated with 9% (95% CI 3, 20%, *p* = 0.017) decrease in the risk of MACE compared with those achieving HbA1c >7.0% (Figure 4A); for every 1% reduction in HbA1c, there was a 15% reduction (95% CI 7%, 29%, *p* <0.001) in the risk of MACE (Figure 4B).

The pattern of the association between HbA1c and MACE was investigated to estimate the existence of a threshold for the loss of benefit. Through polynomial meta-regression, we identified linear regression as the best fit for the association between MACE and the level of HbA1c or the magnitude of the change in HbA1c after therapy, which does not suggest the existence of U or J curves for this association up to HbA1c levels between 6.5 and 7.0%. HbA1c levels ≤ 6.5% were not found in the RCTs.

Among RCTs that reported post-treatment values of HbA1c ≤ 7.0%, we found more frequently studies that included patients with shorter T2DM duration (*p* = 0.02), with longer follow-up (*p* = 0.01) and with GLP1-RA as their active arm (*p* = 0.03). These three variables were independently associated with the risk of developing MACEs. To circumvent these limitations, we performed multivariate meta-regressions for the risk of MACE adjusting for these covariates and, as shown in Table 2, each 1% decrease in HbA1c was associated with an HR for MACE of 0.90 (95% CI 0.75, 0.98, *p* = 0.011, I^2^ = 0%) among RCTs with SGLT2i, DPP4i, pioglitazone, or GLP1-RA in the active arm. Likewise, the association between HbA1c ≤ 7.0% after treatment was also associated with an HR for MACE of 0.83 (95% CI 0.71, 0.95, *p* = 0.009, I^2^ = 0%) in the adjusted analysis.

### Sensitivity Analyses

Sensitivity analyses were conducted using three approaches: (i) the exclusion of one study with the largest exposures (sample size ^*^ follow-up time) per drug in the active arm, (ii) the exclusion of two studies per drug group with the largest exposures, and (iii) the exclusion of ACCORD, ADVANCE, and VADT trials. The first two approaches address the impact of potential class-related mechanisms on the relationship between achieved HbA1c and the risk of MACE, and the third approach addresses the potential influence of the use of the old therapies with a higher risk of severe hypoglycemia.

To exclude that the largest trials are critical to results presented as main analyses, in the first sensitivity analyses we excluded the trials TECOS, REWIND, DECLARE, SCORED and Dormandy (24). As shown in Supplementary Figure S2A, in an additive model network meta-analysis with random-effects, only SGLT2i alone and GLP1-RA alone and the associations SGLT2i + GLP1-RA and SGLT2i + DPP4i were associated with reduced risk of MACE compared to placebo with HRs of 0.85 (95% CI 0.79, 0.92, *p* <0.0001), 0.89 (95% CI 0.85, 0.94, *p* <0.0001), 0.76 (95% CI 0.69, 0.83, *p* <0.0001) and 0.84 (95% CI 0.76, 0.93, *p* = 0.001), respectively, and no heterogeneity (I^2^ = 0%).

In the second sensitivity analysis, we excluded the TECOS, CAROLINA, REWIND, EXCEL, DECLARE, CANVAS, SCORED and Dormandy (24), and the results were unchanged compared to the first sensitivity analysis, and no heterogeneity was found (I^2^ = 0%) (Supplementary Figure S2B). A meta-regression based on this second approach yielded similar results when compared with the pre-exclusion dataset. There were significant relationships between the risk of MACE and the achieved HbA1c levels in the active arm or the differential change in HbA1c in the active arm compared to the control (Table 3).

**Table 3:** Sensitivity analyses^¥^ using meta-regression models for the hazard ratio of MACE as dependent variable.

	**HR**	**95% CI**		***p***
		**Lower bound**	**Upper bound**	
**Bivariate analyses**				
Achieved HbA1c at study end in the active arm (HbA1c ≤ 7.0% vs. HbA1c > 7.0%) vs. MACE
All trials^‡^ (132 study arms)	0.9560	0.7703	1.1865	0.680
SGLT2i, DPP4i, Pioglitazone or GLP1-RA in the active arm (62 study arms^*^)	0.8033	0.6338	0.9773	0.031
				
Change in HbA1c in the active arm compared to control (each reduction of 1%) vs MACE
All trials^ø^ (132 study arms)	0.9036	0.7300	1.0526	0.215
SGLT2i, DPP4i, Pioglitazone or GLP1-RA in the active arm^◇^ (62 study arms^*^)	0.8265	0.6769	0.9581	0.009
Multivariate analyses^**^			
		Lower bound	Upper bound	
Achieved HbA1c at study end in the active arm (HbA1c ≤ 7.0% vs. HbA1c > 7.0%) vs. MACE
All trials^‡‡^ (109 study arms^§^)	0.8598	0.7082	1.0439	0.073
SGLT2i, DPP4i, Pioglitazone or GLP1-RA in the active arm (49 study arms^*^^§^)	0.8328	0.7168	0.8755	0.004
Change in HbA1c in the active arm compared to control (each reduction of 1%) vs MACE
All trials^øø^ (109 study arms^§^)	0.8837	0.6765	1.0402	0.107
SGLT2i, DPP4i, Pioglitazone or GLP1-RA in the active arm^◇◇^ (49 study arms^*^^§^)	0.8194	0.6887	0.9695	0.028

* *Excluding RCTs with outlier HRs for MACE, defined as HR ≥ 2.0 or HR ≤ 0.5*.

** *Adjusted for Time since the diagnosis of T2DM, follow-up time and type of ADT in the active arm. Adjusting variables were selected for their association with HR for MACE in bivariate analyses*.

§ *Some RCTs had missing data for covariates*.

¥ *Data corresponds to sensitivity analysis 2, which excluded the following RCTs: TECOS, CAROLINA, SCORED, REWIND, EXCEL, DECLARE, CANVAS, and Dormandy (24)*.

‡ *I^2^: 3.87%, QE = 90.0 (p = 0.99); tau2: 0.010 (SE = 0.022)*.

*I^2^: 5.31%, QE = 8.7 (p = 0.37); tau2: 0.010 (SE = 0.022)*.

ø *I^2^: 22.08%, QE = 91.3 (p = 0.99); tau2: 0.010 (SE = 0.022)*.

◇ *I^2^: 5.31%, QE = 8.4 (p = 0.35); tau2: 0.010 (SE = 0.022)*.

‡‡ *I^2^: 0%, QE = 70.1 (p = 0.94); tau2: 0.014 (SE = 0.012)*.

*I^2^: 0%, QE = 5.6 (p = 0.21); tau2: 0.010 (SE = 0.021)*.

øø *I^2^: 0%, QE = 83.7 (p = 0.97); tau2: 0.018 (SE = 0.027)*.

◇◇ *I^2^: 0%, QE = 6.1 (p = 0.28); tau2: 0.008 (SE = 0.028)*.

In the third sensitivity analysis, we found no significant changes in the relationship between the risk of MACE and the achieved HbA1c levels in the active arm or the differential change in HbA1c when we excluded the ACCORD, ADVANCE, and VADT trials (data not shown).

## Discussion

The present systematic review and meta-analysis evaluated 126 RCTs with over 740,000 individuals-years and showed that the absolute change in HbA1c and the target ≤ 7.0% were associated with reduced risk of MACE in therapies based on SGLT2i, DPP4i, pioglitazone, or GLP1-RA, with no evidence of increasing all-cause mortality.

Our results are in line with previous meta-analyses (14, 18, 25) and showed that the absolute change in HbA1c and an achieved HbA1c ≤ 7.0% in patients with T2DM is associated with mild reductions in MACE risk. In disagreement with our findings, Wang et al. (25) pooled 15 studies with 88,266 type 2 diabetes individuals and reported that an HbA1c <7.0% did not improve cardiovascular outcomes. It is worth mentioning that their meta-analysis yielded high heterogeneity and pooled various study designs, including trials with post-acute coronary syndromes such as DIGAMI-1 (26). Our differential approach in this meta-analysis was to carry out a more comprehensive literature search and, therefore, with greater statistical power and evaluated SGLT2i, DPP4i, pioglitazone, and GLP1-RA independently.

Our findings are also in line with the results of RCTs designed to investigate the cardiovascular effects of achieving HbA1c ≤ 7.0% such as ACCORD, VADT, and ADVANCE (4–6). These trials randomized individuals for placebo or intense glucose-lowering with insulin/sulfonylurea-based regimens, and intense glucose control did not decrease the incidence of macrovascular events. By analyzing 139 RCTs with all ADTs, the results of our meta-regression do not support a role of achieving HbA1c <7.0% in the incidence of MACE. This was only true specifically for SGLT2i, DPP4i, pioglitazone, and GLP1-RA (69 trials), which suggests insulin and sulfonylurea-based regimens alter the relationship between glucose-lowering (achieved HbA1c) and the risk of MACE. There are several explanations for these findings, including that insulin and sulfonylurea-based regimens increase the risk of weight gain and severe hypoglycemia. The adverse effects have negative cardiovascular effects that could jeopardize any potential benefit of glucose-lowering. In a systematic review and meta-analysis with 903,510 individuals (10), severe hypoglycemia was associated with a higher risk of cardiovascular disease (relative risk 2.05, 95% confidence interval 1.74–2.42; *p* <0.001). Severe hypoglycemia episodes are dose-dependently associated with increased risk for myocardial infarction, as well as all-cause mortality, stroke, and heart failure (9). Risks for all outcomes were highest within 1 year from the index date and showed decreasing trends with follow-up (9). From a pathophysiological standpoint, hypoglycemic events trigger inflammation, catecholamine release, and sympathetic activation, prompting a prothrombotic environment, increasing blood viscosity and promoting platelet activation/aggregation, leukocyte mobilization and coagulation (27, 28). Together, these mechanisms lead to endothelial dysfunction, decreased vasodilation, arrhythmias and increased cardiac workload, contributing to the risk of cardiovascular events (13).

Notably, although our meta-analysis did not capture significant heterogeneity among RCTs regarding the risk of MACE within ADT classes, there was a marked imbalance in baseline characteristics of individuals enrolled in RCTs with HbA1c ≤ 7.0% compared to RCTs that achieved HbA1c >7.0%. RCTs achieving HbA1c >7.0% were shorter in duration and more frequently enrolled individuals with long-term T2D (>8–10 years of disease). Although this is expected, no prior meta-analysis adjusted the regressions for these important cofactors (18, 25). Hence, in this study, we performed a step forward using multivariate meta-regression analyses adjusting for T2DM duration, follow-up duration, and the effect of treatment in the active arm, and confirmed that achieving HbA1c ≤ 7.0% with SGLT2i, GLP1-RA, DPP4i, or pioglitazone was associated with a decreased risk of MACE compared to >7.0%.

Some findings from this meta-analysis indicate a potential contribution of blood glucose lowering in the reduction of macrovascular events. With SGLT2i, DPP4i, pioglitazone or GLP1-RA, RCTs with post-therapy HbA1c ≤ 7.0% were consistently associated with a 9% lower risk of MACE compared with RCTs that achieved HbA1c >7.0%, regardless of the therapies used. A linear trend was found between MACE and HbA1c in the range of 6.5–8.0%, with no evidence of U- or J-shaped curves and in a magnitude of association that is similar to that reported between hyperglycemia and MACE in observational studies (1, 2). We plan to verify this association by a meta-regression exclusively based on DPP4i, whose RCTs demonstrated a low risk of severe hypoglycemia, no weight gain, and no direct cardiovascular benefit. However, the sample size with the combination of these RCTs did not provide sufficient statistical power for this analysis. We found a very similar effect of SGLT2i, GLP1RA, and pioglitazone in the decrease of MACE risk. As commented above, these effects result from a wide range of mechanisms, which are concomitant and difficult to dissociate from their glucose-lowering effects. Thus, although this meta-analysis indicates the existence of a direct effect of lowering blood glucose levels on the incidence of MACE, our data do not allow us to determine the exact size of this effect.

Our study had limitations. First, our results were obtained by meta-regression analysis from RCTs, which is inferior to analyses at the patient level. Nevertheless, in sensitivity analyses, when we evaluated different scenarios, excluding trials with larger exposures, we noticed similar results. Second, we did not include RCTs specifically designed to heart failure with or without overt diabetes such as EMPEROR-Reduced and EMPEROR-Preserved since their addition would add a complex bias to our analysis. Third, as mentioned above, SGLT2i, pioglitazone and GLP1-RA have demonstrated MACE risk-reduction mechanisms that are independent of glycemic control or hypoglycemia. Therefore, the available data do not allow us to accurately estimate the magnitude of the effect of lowering blood glucose levels in reducing MACE.

In summary, more intense reductions in HbA1c and lower levels of HbA1c achieved with SGLT2i, DPP4i, pioglitazone and GLP1-RA are associated with a reduced risk of MACE. Targeting HbA1c between 6.5 and 7% with SGLT2i, GLP1-RA, pioglitazone, or DPP4i may be associated with cardiovascular risk reduction in light of the available RCT evidence.

## Data Availability Statement

The original contributions presented in the study are included in the article/Supplementary Material, further inquiries can be directed to the corresponding author.

## Author Contributions

LC, AN, IB, BL, AB, CC, JLB, JB, and AS made a substantial contribution to the conception and design of the work and manuscript drafting. AN, IB, BL, AB, CC, JLB, and JB contributed to the acquisition, analysis, and interpretation of the data. All authors were involved in drafting and revision of the manuscript for important intellectual content and approved the final version to be published.

## Funding

AS was supported by a Research Career Awards grant from the Brazilian National Research Council (CNPq) (grant number 304257/2021-4).

## Conflict of Interest

The authors declare that the research was conducted in the absence of any commercial or financial relationships that could be construed as a potential conflict of interest.

## Publisher's Note

All claims expressed in this article are solely those of the authors and do not necessarily represent those of their affiliated organizations, or those of the publisher, the editors and the reviewers. Any product that may be evaluated in this article, or claim that may be made by its manufacturer, is not guaranteed or endorsed by the publisher.
