# Supplementary material for: Glucose-Lowering and the Risk of Cardiovascular Events With Antidiabetic Therapies: A Systematic Review and Additive-Effects Network Meta-Analysis
Source: Front Cardiovasc Med. 2022 Apr 29;9:876795. doi: 10.3389/fcvm.2022.876795 (PMC9098935; doi:10.3389/fcvm.2022.876795)
Supplement: Supplementary file 1 [file Data_Sheet_2.PDF]

SUPPLEMENTARY MATERIAL

Glucose-Lowering and the Risk of Cardiovascular Events with Novel Antidiabetic Therapies: A Systematic Review and Additive-effects Network Meta-Analysis

Luiz Sergio Fernandes de Carvalho MD MSc PhD[a,b,c], Ana Claudia Cavalcante Nogueira MD MSc[b,c], Isabella Bonilha PhD[c], Beatriz Luchiari[c], Alexander Benchimol MD PhD[d], Carlos Eduardo Barra Couri MD PhD[d,e], Jairo Lins Borges MD PhD[d,e], Joaquim Barreto MD[c], Andrei Carvalho Sposito MD PhD[c,*]

[a] Laboratory of Data for Quality of Care and Outcomes Research, Clarity Healthcare Intelligence, Jundiaí, SP, Brazil.

[b] Catholic University of Brasília (UCB), Brasilia, DF, Brazil.

[c] Atherosclerosis and Vascular Biology Laboratory (Atherolab), Cardiology Division, University of Campinas (Unicamp), Campinas, SP, Brazil.

[d] State Institute of Diabetes and Endocrinology, Rio de Janeiro, RJ, Brazil;

[e] Department of Hematology, Rio de Janeiro Federal University, Rio de Janeiro, RJ, Brasil;

[f] São Paulo Federal University (UNIFESP), São Paulo, SP - Brazil.

*** Correspondence:**

Andrei C Sposito

andreisposito@gmail.com

Table S1. Baseline characteristics of patients in the 126 randomized controlled trials (143 study arms)

RCT	Active arm	Control arm	Follow-up time (years, mean)	Sample size	Weight	Exposure (patient-years)	Events patient-years (Active arm)	Events patient-years (Control arm)	Women (%, mean)	Age (years, mean)	Time since DM diagnosis (years)	HbA1c at baseline (%, mean±SD)		
ACCORD (2008)(1)	Sulfonylurea+Insulin	Sulfonylurea+Insulin	3.50	10251	4,97%	35879	19.61	20.69	38.7	62.2	10	8.30	±	0.90
ADVANCE (2008)(2)	Sulfonylurea+Insulin	Sulfonylurea+Insulin	5.00	11140	7,72%	55700	20	21.19	42.4	66.00	8.00	7.52	±	1.40
AWARD-3(3)	GLP1A	Metformin	0.96	807	0,11%	775	3.87	0	55.00	55.00	3.00	7.60	±	0.80
AWARD-5(4)	GLP1A	DPP4i	1.90	921	0,24%	1750	5.21	6.68	52.00	54.00	7.00	8.10	±	1.10
AWARD-9(5)	GLP1A	Placebo	0.53	300	0,02%	159	25.16	0	41.30	60.60	13.30	8.30	±	0.80
Abdul-ghani (2017) (6)	GLP1A+TZD	Insulin	1.00	231	0,03%	231	0	46.30	63.00	52.00	10.90	10	±	0.50
Arechavaleta (2010) (7)	DPP4i	Sulfonylurea	0.58	1035	0,08%	600	0	6.64	53.80	56.20	6.70	7.50	±	0.80
Arjona (2013) (8)	DPP4i	Sulfonylurea	1.00	423	0,06%	423	23.70	23.58	45.10	64.30	10.10	7.80	±	0.70
Bailey (2013) (9)	SGLT2i	Placebo	2.00	546	0,15%	1092	7.33	14.60	45.00	53.70	5.80	8.11	±	0.96
Bailey (2015) (10)	SGLT2i	Placebo	2.00	274	0,08%	548	5.03	0	58.70	52.70	2.10	7.84	±	0.87
Barnett (2013) (11)	DPP4i	Placebo	0.96	455	0,06%	437	6.85	6.90	55.00	57.30	12.20	8.60	±	0.90
Barnett (2014) (12)	SGLT2i	Placebo	1.00	374	0,05%	374	5.35	5.35	43.30	65.10		8.09	±	0.80
Bode (2015) (13)	SGLT2i	Placebo	1.96	714	0,19%	1399	9.63	6.46	39.70	63.20	11.40	7.80	±	0.80

Bolli (2009) (14)	DPP4i	SGLT2i	0.96	576	0,08%	553	7.06	11.12	35.90	57.00	6.40	8.40	±	0.90
Bosi (2007) (15)	DPP4i	Placebo	0.46	544	0,03%	250	6.01	0	46.90	54.50	6.20	8.30	±	0.90
Bosi (2011) (16)	DPP4i	SGLT2i	0.96	803	0,11%	771	5.16	7.83	48.90	55.90	6.90	8.10	±	0.83
Buse (2011) (17)	GLP1A+Insulin	Insulin+Placebo	0.57	259	0,02%	148	0	14.38	36.00	59.00	12.00	8.50	±	0.96
CANVAS(18)	SGLT2i	Placebo	3.50	10142	4,92%	35497	26.62	30.96	36.70	63.40	13.70	8.20	±	0.90
CARMELINA(19)	DPP4i	Placebo	2.20	6979	2,13%	15354	56.46	54.78	35.70	65.60	14.50	8.00	±	1.00
CAROLINA(20)	DPP4i	Sulfonylurea	6.30	6033	5,27%	38008	18.69	19.09	40.80	64.20	6.20	7.20	±	0.60
CONFIDENCE (1) (21)	GLP1A	Insulin	0.92	224	0,03%	206	9.88	9.53	38.60	51.00		8.20		
CONFIDENCE (2) (21)	GLP1A	Pioglitazone	0.92	228	0,03%	210	9.88	9.21	44.90	50		8.00		
Cefalu (2015) (22)	SGLT2i	Placebo	0.96	914	0,12%	877	13.74	6.81	31.40	63.00	12.30	8.08	±	0.80
Chacra (2011) (23)	DPP4i	Placebo	1.40	769	0,15%	1077	11.38	10.70	53.90	55.10	6.80	8.40	±	0.90
Charpentier (2009) (24)	Pioglitazone	Placebo	0.58	289	0,02%	168	12.08	0	35.00	60.20	12.50	8.10	±	0.70
Chen (2018) (25)	GLP1A	Sulfonylurea	0.50	720	0,05%	360	4.18	0	46.30	52.00	3.80	7.90	±	1.01
Davies (2021) (26)	GLP1A	Placebo	1.25	1210	0,21%	1512	8.8	2.4	49.95	55.25	8.1	8.10	±	0.80
DECLARE(27)	SGLT2i	Placebo	4.20	17160	9,99%	72072	20.97	22.29	37.90	64.00	10	8.30	±	1.20
DUAL IV(28)	GLP1A+Insulin	Placebo	0.50	435	0,03%	218	13.84	0	50	59.40	9.30	7.90	±	0.60

DURATION-2 (1) (29)	GLP1A	DPP4i	0.50	326	0,02%	163	0	12.05	48.00	52.00	5.00	8.50	±	1.20
DURATION-2 (2) (29)	GLP1A	Pioglitazone	0.50	325	0,02%	163	0	36.36	52.00	53.00	6.00	8.50	±	1.10
DURATION-4 (1) (30)	GLP1A	Metformin	0.50	494	0,03%	247	0	8.13	37.40	54.00	2.60	8.60	±	1.20
DURATION-4 (2) (30)	GLP1A	TZD	0.50	411	0,03%	206	0	12.27	40.50	55.00	2.70	8.50	±	1.20
DURATION-7(31)	GLP1A+Insulin	Insulin+Placebo	0.53	461	0,03%	244	0	8.20	53.50	57.60	11.10	8.53	±	0.92
DURATION-8 (1)(32)	GLP1A	SGLT2i	0.96	457	0,06%	439	22.94	18.12	52.20	54.50	7.10	9.30	±	1.00
DURATION-8 (2) (32)	GLP1A+SGLT2i	SGLT2i	0.96	458	0,06%	440	9.14	18.12	52.20	54.50	7.10	9.30	±	1.00
DURATION-8 (3) (32)	GLP1A+SGLT2i	GLP1A	0.96	455	0,06%	437	9.14	22.94	48.90	54.20	7.40	9.30	±	1.10
DeFronzo (2009) (33)	DPP4i	Placebo	0.46	743	0,05%	342	0	12.14	46.40	54.80	6.70	8.10	±	0.90
Del Prato (2014) (34)	DPP4i	Sulfonylurea	1.92	2639	0,70%	5067	4.13	6.56	49.50	55.40	5.50	7.60	±	0.62
Del Prato (2015) (34)	SGLT2i	Sulfonylurea	4.00	801	0,44%	3204	0.63	2.49	45.10	58.60	6.60	7.74	±	0.89
Dormandy (2005) (35)	Pioglitazone	Placebo	2.88	5238	2,09%	15059	44.33	51.12	34.00	61.60	8.00	7.90		
ELIXA(36)	GLP1A	Placebo	2.08	6068	1,75%	12621	62.91	61.64	30.90	60.60	9.40	7.60	±	1.30
EMPA-REG(37)	SGLT2i	Placebo	3.10	7020	3,02%	21762	33.72	38.99	28.00	63.20		8.08	±	0.80
EXAMINE(38)	DPP4i	Placebo	1.50	5380	1,12%	8070	75.28	78.64	32.00	60.70	9.18	8.00	±	1.10
EXSCEL (39)	GLP1A	Placebo	3.20	14752	6,54%	47206	35.64	38.24	38.00	62.00	12.00	8.00		

Ferdinand (2014) (40)	GLP1A	Placebo	0.50	755	0,05%	378	3.96	8.00	47.60	56.40	8.40	7.90	±	0.80
Ferrannini (2009) (41)	Sulfonylurea	DPP4i	1.00	2789	0,39%	2789	15.79	8.60	47.20		5.71	7.31	±	0.64
GET-GOAL(42)	GLP1A	Placebo	1.40	482	0,09%	675	6.65	4.46	55.00	58.20	6.20	8.00	±	0.80
Gallwitz (2012) (43)	DPP4i	Sulfonylurea	2.00	1551	0,43%	3102	7.09	14.84	39.00	59.80		7.70	±	0.90
Gantz (2017) (44)	DPP4i	Placebo	1.80	4202	1,05%	7564	30.16	30.13	29.30	63.60	12.10	8.00	±	0.90
GetGoal-Duo-1(45)	GLP1A	Placebo	0.46	446	0,03%	205	9.75	19.50	51.00	56.00	9.60	7.60	±	0.50
GetGoal-L(46)	GLP1A	Placebo	0.50	495	0,03%	248	18.29	35.93	51.00	57.00	12.40	8.40	±	0.80
GetGoal-L-Asia(47)	GLP1A	Placebo	0.46	311	0,02%	143	28.23	0	49.00	58.00	14.10	8.52	±	0.78
GetGoal-M-Asia(48)	GLP1A	Placebo	0.46	390	0,02%	179	22.18	11.21	53.10	55.10	6.80	7.85	±	0.71
GetGoal-O (49)	GLP1A	Placebo	0.46	350	0,02%	161	0	37.48	48.30	74.40	14.60	8.05	±	0.70
GetGoal-S(50)	GLP1A	Placebo	0.46	859	0,05%	395	30.30	30.51	47.40	57.80	9.80	8.20	±	0.80
Giles (2008) (51)	Pioglitazone	Sulfonylurea	0.50	518	0,04%	259	68.70	62.50	23.00	63.40	11.71	8.95		
Giles (2010) (52)	Pioglitazone	Sulfonylurea	1.00	300	0,04%	300	0	13.42	44.00	64.00		8.30		
Goke (2008) (53)	DPP4i	Metformin	2.00	463	0,13%	926	1.64	0	39.00	54.08	2.20	8.80	±	0.10
Goke (2013) (54)	DPP4i	Sulfonylurea	1.90	858	0,23%	1630	3.69	7.34	46.00	57.60	5.40	7.65	±	0.04
HARMONY(55)	GLP1A	Placebo	1.50	9463	1,97%	14195	47.63	60.30	31.00	64.20	14.20	8.72	±	1.50

HARMONY-1(56)	GLP1A	Placebo	2.90	301	0,12%	873	4.60	2.28	41.70	54.90	7.90	8.10	±	0.90
HARMONY-3 (1) (57)	GLP1A	Sulfonylurea	1.90	609	0,16%	1157	12.20	3.43	48.50	54.40	6.00	8.10	±	0.80
HARMONY-3 (2) (57)	GLP1A	DPP4i	1.90	604	0,16%	1148	12.20	3.49	54.00	54.30	5.80	8.10	±	0.80
HARMONY-3 (3) (57)	GLP1A	Placebo	1.90	403	0,11%	766	12.20	15.63	50.50	56.10	6.70	8.20	±	0.90
HARMONY-5 (1) (58)	GLP1A	Pioglitazone	2.90	548	0,22%	1589	1.27	7.47	46.60	55.70	9.20	8.29	±	0.88
HARMONY-5 (2) (58)	GLP1A	Placebo	2.90	386	0,16%	1119	1.27	9.00	39.10	55.70	9.30	8.26	±	0.98
HARMONY-8(59)	GLP1A	DPP4i	0.96	495	0,07%	475	20.92	29.64	47.20	63.50	11.62	8.23	±	0.94
Haering (2015) (60)	SGLT2i	Placebo	1.40	666	0,13%	932	6.48	12.70	50.20	56.90		8.20	±	0.80
Handelsman (2017) (61)	DPP4i	Sulfonylurea	1.00	751	0,10%	751	7.98	5.33	43.70	58.00	7.70	7.40	±	0.70
Henry (2013)-1(62)	DPP4i	Pioglitazone	1.04	751	0,11%	781	10.34	3.40	43.50	51.15	3.76	8.80	±	1.10
Henry (2013)-2(62)	DPP4i	DPP4i+ Pioglitazone	1.04	767	0,11%	798	10.34	6.62	43.50	53.40	3.97	8.80	±	1.10
Henry (2013)-3(62)	DPP4i+TZD	Pioglitazone	1.04	1146	0,17%	1192	6.62	3.40	43.50	51.15	3.76	8.80	±	1.10
Hollander (2011) (63)	DPP4i	Placebo	1.40	565	0,11%	791	9.37	3.88	53.80	54.00	5.10	8.20	±	1.10
Hollander (2019) (64)	SGLT2i	Sulfonylurea	2.00	1305	0,36%	2610	9.77	5.75	48.50	57.90	7.60	7.80	±	0.60
Hong (2013) (65)	Sulfonylurea	Metformin	3.00	304	0,13%	912	72.07	49.15	21.80	62.80	5.60	7.60	±	1.70
IDegLira(66)	GLP1A+Insulin	Insulin	0.50	398	0,03%	199	10.05	20.10	47.00	58.00	11.00	8.80	±	0.70

Jain (2006) (67)	Pioglitazone	Sulfonylurea	1.00	502	0,07%	502	7.97	11.95	43.80	52.10	9.40	9.20	±	1.26
Ji (2021) (68)	GLP1	DPP4	0.55	868	0,07%	477	1.8	0	40.9	63.00	6.30	8.1	±	0.9
Kawamori (2018) (69)	SGLT2i	Placebo	0.96	275	0,04%	264	5.72	11.20	22.60	59.80	8.70	8.36	±	0.74
Kohan (2014) (70)	SGLT2i	Placebo	1.96	252	0,07%	494	15.18	36.44	36.90	67.00	15.70	8.53	±	1.28
Kovacs (2015) (71)	SGLT2i	Placebo	1.40	498	0,10%	697	8.58	12.99	55.80	54.60		8.16	±	0.92
LEADER(72)	GLP1A	Placebo	3.80	9340	4,92%	35492	34.28	39.09	36.00	64.40	12.90	8.70	±	1.50
LIRA-RENAL(73)	GLP1A	Placebo	0.50	277	0,02%	139	57.14	14.60	52.60	66.30	14.20	8.00	±	0.85
LIRA-SWITCH(74)	GLP1A	DPP4i	0.50	406	0,03%	203	0	9.80	39.00	56.50	7.60	8.20		
Laakso (2015) (75)	DPP4i	Sulfonylurea	1.00	241	0,03%	241	8.47	48.78	35.20	65.90		8.03	±	0.94
Leiter (2014) (76)	SGLT2i	Placebo	0.96	962	0,13%	924	13.02	12.97	33.00	63.60	13.00	8.10	±	0.80
Leiter (2015) (77)	SGLT2i	Sulfonylurea	2.00	1450	0,40%	2900	5.68	5.19	45.40	56.30	6.60	7.80	±	0.80
Lewin (2015) (78)	SGLT2i	DPP4i	1.00	398	0,06%	398	3.77	15.04	43.60	53.80		8.05	±	0.89
Lewin (2015)-1(78)	SGLT2i+DPP4i	DPP4i	1.00	402	0,06%	402	7.43	15.04	43.60	53.80		8.05	±	0.89
Lewin (2015)-2(78)	SGLT2i	DPP4i	1.00	398	0,06%	398	3.77	15.04	43.60	53.80		8.05	±	0.89
Mathieu (2016) (79)	SGLT2i+DPP4i	Placebo	1.00	320	0,04%	320	6.25	0	52.50	55.00	8.00	8.17	±	0.98
Matthews (2010) (80)	DPP4i	Sulfonylurea	0.96	3118	0,41%	2993	10	8.70	46.10	57.50	5.70	7.30	±	0.70

Matthews (2019) (81)	DPP4i	Placebo	5.00	2001	1,39%	10005	4.81	6.58	51.30	54.60	0.28	6.70	±	0.50
Mazzone (2006) (82)	Pioglitazone	Sulfonylurea	1.50	458	0,10%	687	0	5.85	37.30	59.90	7.50	7.40	±	0.97
Merker (2015) (83)	SGLT2i	Placebo	1.40	637	0,12%	892	4.98	13.80	44.00	56.00		7.90	±	0.70
Moses (2015) (84)	DPP4i	SGLT2i	1.00	422	0,06%	422	4.76	0	53.80	55.40	8.00	8.40	±	0.90
Muller-Wieland (2018)-1(85)	SGLT2i	Sulfonylurea	0.96	627	0,08%	602	6.63	3.33	33.50	58.60	6.70	8.30	±	0.80
Muller-Wieland (2018)-2(85)	SGLT2i+DPP4i	Sulfonylurea	0.96	625	0,08%	600	3.34	3.33	33.50	58.60	6.70	8.30	±	0.80
Muller-Wieland (2018)-3(85)	SGLT2i	Sulfonylurea	0.96	939	0,12%	901	6.66	0	33.50	58.60	6.70	8.30	±	0.80
Nauck (2016)-1(86)	DPP4i+TZD	Pioglitazone	0.63	801	0,07%	501	8.16	11.74	47.00	56.60		8.13	±	0.90
Nauck (2016)-2(86)	DPP4i+TZD	DPP4i	0.63	527	0,05%	329	8.16	23.70	38.00	56.00		8.00	±	0.90
Nissen (2008) (87)	Pioglitazone	Sulfonylurea	1.50	543	0,11%	815	12.35	14.65	34.10	59.70		7.40	±	1.00
Olansky (2011) (88)	DPP4i	Metformin	0.81	1246	0,14%	1009	1.98	0	43.00	50	3.20	9.80	±	1.80
PIONEER-3(89)	GLP1A	DPP4i	1.40	1863	0,36%	2608	8.19	4.59	49.00	58.00	8.80	8.30	±	0.90
PIONEER-6(90)	GLP1A	Placebo	1.30	3183	0,57%	4138	29.49	36.72	31.40	66.00	15.10	8.20	±	1.60
PIONEER-8(91)	GLP1A	Placebo	0.96	731	0,10%	702	17.14	22.64	42.90	60	14.80	8.20	±	0.70
Perez (2010)-2 (92)	Pioglitazone	Metformin	0.50	399	0,03%	200	10.58	0	53.00	53.70		8.65	±	0.07

Perkovic (2019) (93)	SGLT2i	Placebo	2.62	4401	1,60%	11531	37.61	46.69	33.30	63.20	16.00	8.30	±	1.30
Pfutzner (2011-A) (94)	DPP4i	Placebo	1.40	1304	0,25%	1826	5.12	6.53	50.30	51.80	1.70	9.40	±	1.30
Pinget (2013) (95)	GLP1A	Placebo	1.40	484	0,09%	678	0	8.87	49.00	55.30	8.10	8.10	±	0.80
Pratley (2018) (96)	SGLT2i	DPP4i	1.00	745	0,10%	745	2.01	0	38.70	54.80	6.20	8.50	±	1.00
REWIND(97)	GLP1A	Placebo	5.40	9901	7,41%	53465	22.23	24.79	46.10	66.20	10.60	7.40	±	1.10
Ridderstrale (2018) (98)	SGLT2i	Sulfonylurea	4.00	1545	0,86%	6180	4.90	8.01	46.00	55.70		7.92	±	0.86
Roden (2015)-1 (99)	SGLT2i	DPP4i	1.50	671	0,14%	1007	7.44	5.98	36.80	55.10		7.85	±	0.79
Roden (2015)-2 (99)	SGLT2i	Placebo	1.50	676	0,14%	1014	7.44	2.92	46.10	54.90		7.91	±	0.78
Rosenstock (2013) (100)	DPP4i	Sulfonylurea	1.00	441	0,06%	441	0	9.13	56.20	69.80	5.94	7.45	±	0.63
Rosenstock (2014) (101)	SGLT2i	Placebo	1.00	563	0,08%	563	5.33	5.32	60	55.30		8.33	±	7.90
Rosenstock (2015)-1(102)	SGLT2i	Placebo	1.50	494	0,10%	741	14.40	11.76	47.00	58.10		8.20	±	0.80
Rosenstock (2019)-1(103)	SGLT2i+DPP4i	SGLT2i	0.46	579	0,04%	266	0	7.52	47.40	55.90	7.60	8.20	±	0.90
SCORED (104)	SGLT2i	Placebo	2.75	10584	4,03%	29106	124.7	160.7	44.90	69		8.3		
SAVOR-TIMI 53(105)	DPP4i	Placebo	2.10	16492	4,80%	34633	35.25	35.31	32.70	65.00	10.30	8.00	±	1.40
SCALE(106)	GLP1A	Placebo	1.03	846	0,12%	871	7.66	13.74	54.20	54.70	6.70	7.90	±	0.80
SUSTAIN-1(107)	GLP1A	Placebo	0.57	387	0,03%	221	6.80	0	46.00	53.90	4.06	7.95	±	0.85

SUSTAIN-6(108)	GLP1A	Placebo	1.90	3297	0,87%	6264	34.49	46.60	41.50	64.80	14.00	8.70	±	1.49
SUSTAIN-8(109)	GLP1A	SGLT2i	0.96	788	0,10%	756	2.64	2.64	49.00	57.50	7.20	8.20	±	1.00
Schernthaner (2013) (110)	SGLT2i	DPP4i	0.96	755	0,10%	725	8.29	5.51	43.10	56.70	9.70	8.10	±	0.90
Schernthaner (2015) (111)	DPP4i	Sulfonylurea	0.96	720	0,10%	691	11.57	8.68	36.70	72.70	7.60	7.62	±	0.65
Schweizer (2009) (112)	DPP4i	Metformin	0.46	335	0,02%	154	12.86	13.10	47.00	70.20	3.00	7.70	±	0.60
Scott (2018) (113)	SGLT2i	SGLT2i	0.46	613	0,04%	282	0	7.08	45.00	67.70	10.50	7.70	±	0.70
Seck (2010) (114)	DPP4i	Sulfonylurea	2.00	1172	0,32%	2344	0.85	3.42	38.70	56.60	6.20	7.60	±	0.90
Seino (2010) (115)	GLP1A	Sulfonylurea	0.46	400	0,03%	184	24.33	49.41	35.00	58.50	8.50	8.78	±	0.97
Seino (2016) (116)	GLP1A+Insulin	Insulin+Placebo	0.69	257	0,02%	177	0	22.30	42.30	59.80	14.69	8.80	±	0.90
Soloist-WHF (2020) (117)	SGLT2i	Placebo	0.75	1222	0,13%	916			33.75	69.00		7.15		
Strojek (2014) (118)	SGLT2i	Placebo	0.90	592	0,07%	533	9.94	7.66	51.00	60.30	7.40	8.15	±	0.80
TECOS(119)	DPP4i	Placebo	3.00	14671	6,10%	44013	33.87	33.88	29.50	65.50	11.60	7.20	±	0.50
Tolman (2009) (120)	Pioglitazone	Sulfonylurea	3.00	2097	0,87%	6291	5.71	8.29	44.50	55.00	5.40	9.50	±	2.00
VADT (2009) (121)	Sulfonylurea+Insulin	Sulfonylurea+Insulin	5.60	1791	1,39%	10030	47.05	52.44	26.00	60.4	11.50	9.40		
Wainstein (2011) (122)	Pioglitazone	DPP4i+Metformin	0.61	517	0,04%	315	0	6.28	45.20	52.40	3.20	9.00	±	1.30
Wilding (2012) (123)	SGLT2i	Placebo	0.90	800	0,10%	720	3.66	0	50.80	58.80	13.50	8.47	±	0.77

Wilding (2013) (124)	SGLT2i	Placebo	1.00	469	0,07%	469	3.19	12.82	51.30	56.80	10.30	8.10	±	0.90
Yale (2014) (125)	SGLT2i	Placebo	1.00	269	0,04%	269	27.93	33.33	36.70	68.20	16.40	8.00	±	0.90
Yang (2018) (126)	SGLT2i	Placebo	0.46	272	0,02%	125	0	16.35	51.90	58.60	12.20	8.58	±	0.81

Continuous data are presented as mean ± SD.

RCTs in red the studies with at least 1% of overall weight, which is based on the ratio between each individual study exposure (in patient-years) vs overall exposure.

Most of large RCTs are noted as their acronyms and other studies are annotated as First author (publication year).

The presence of dash followed by a number (e.g. Rosenstock (2015)-1) denotes that one of multiple study arms is included.

This table excluded studies or study arms in which no clinical event occurred in the active or in the control arm.

Table S2. Risk of bias analysis

Study	Random sequence generation	Allocation concealment	Blinding of participante and personnel	Blinding of outcome assessment	Incomplete outcome data	Selective reporting	Other bias
ACCORD	+	+	+	+	+	+	+
ADVANCE	+	+	+	+	+	+	+
AWARD-3	+	+	+	+	+	+	+
AWARD-5	+	+	+	+	+	+	+
AWARD-9	+	+	+	+	+	+	+
Abdul-ghani [2017]	+	+	−	−	+	+	+
Arechavaleta [2010]	+	+	+	+	+	+	+
Arjona [2013]	+	+	+	+	+	+	+
Bailey [2013]	+	+	+	+	+	+	+
Bailey [2015]	+	+	+	+	+	+	+
Barnett [2013]	+	+	+	+	+	+	+
Barnett [2014]	+	+	+	+	+	+	+
Bode [2015]	+	+	+	+	+	+	+
Bolli [2009]	+	+	+	+	?	+	+
Bosi [2007]	+	+	+	+	+	+	+
Bosi [2011]	+	+	+	+	+	+	+
Buse [2011]	+	+	+	+	+	+	+
CANVAS	+	+	+	+	+	+	+
CARMELINA	+	+	+	?	+	+	+
CAROLINA	+	?	+	+	+	+	+
CONFIDENCE	+	+	−	−	+	+	+
Cefalu [2015]	?	?	+	+	+	+	+
Chacra [2011]	+	+	+	+	+	+	+
Charpentier [2009]	?	?	+	+	+	+	+
Chen [2018]	+	+	+	+	+	+	+
Davies [2021]	+	+	+	+	+	+	+
DECLARE	+	+	+	+	+	+	+
DUAL IV	+	+	+	+	+	+	+
DURATION-2	+	+	+	+	+	+	+
DURATION-4	+	+	+	+	+	+	+
DURATION-7	+	+	+	+	+	+	+
DURATION-8	+	+	+	+	+	+	+
DeFronzo [2009]	+	+	+	+	+	+	+
Del Prato [2015]	?	?	+	+	+	+	+
Del Prato [2014]	+	+	+	+	+	+	+
Dormandy [2005]	+	+	+	+	+	+	+
ELIXA	+	+	+	+	?	+	+
EMPA-REG	+	+	+	+	+	+	+
EXAMINE	?	?	+	+	?	+	+
EXSCEL	+	?	+	+	?	+	+
Ferdinand [2014]	+	+	+	+	+	+	+
Ferrannini [2009]	?	?	+	+	+	+	+
GET-GOAL	?	?	+	+	+	+	+
Gallwitz [2012]	+	+	+	+	+	+	+
Gantz [2017]	+	?	+	+	?	+	+
GetGoal-Duo-1	+	+	+	+	+	+	+
GetGoal-L	+	+	+	+	+	+	+
GetGoal-L-Asia	+	+	+	+	+	+	+
GetGoal-M-Asia	+	+	+	+	?	+	+
GetGoal-O	+	+	+	+	+	+	+
GetGoal-S	?	+	+	?	?	+	+
Giles [2008]	?	?	+	+	+	+	+
Giles [2010]	?	?	+	+	+	+	+
Goke [2008]	?	?	+	+	?	+	+
Goke [2013]	+	+	+	+	+	+	+
HARMONY	+	+	+	+	+	+	+
HARMONY-1	+	+	+	+	+	+	+
HARMONY-3	?	+	+	+	+	+	+
HARMONY-5	+	+	+	+	+	+	+
HARMONY-8	+	+	+	+	+	+	+
Haering [2015]	+	+	+	+	?	+	+
Handelsman [2017]	+	+	+	+	+	+	+
Henry [2013]	+	+	+	+	+	+	+
Hollander [2011]	?	+	+	+	+	+	+
Hollander [2019]	+	+	+	+	+	+	+
Hong [2013]	+	?	+	?	?	+	+
IDegLira	+	+	+	+	+	+	+
Jain [2006]	?	+	+	?	+	+	+
Ji [2021]	+	+	+	+	+	+	+
Kawamori [2018]	+	+	+	+	+	+	+
Kohan [2014]	?	+	+	+	+	+	+
Kovacs [2015]	+	+	+	+	+	+	+
LEADER	?	+	+	+	?	+	+
LIRA-RENAL	+	+	+	+	+	+	+
LIRA-SWITCH	+	+	+	?	?	+	+
Laakso [2015]	?	+	+	?	+	+	+
Leiter [2014]	+	+	+	+	?	+	+
Leiter [2015]	+	+	+	+	+	+	+
Lewin [2015]	+	+	+	+	+	+	+
Mathieu [2016]	+	+	+	+	+	+	+
Matthews [2010]	?	?	+	+	+	+	+
Matthews [2019]	+	+	+	+	+	+	+
Mazzone [2006]	?	?	+	+	+	+	+
Merker [2015]	+	+	+	+	+	+	+
Moses [2015]	?	+	+	+	+	+	+
Muller-Wieland [2018]	+	+	+	+	+	+	+
Nauck [2016]	+	+	+	+	+	+	+
Nissen [2008]	+	+	+	+	+	+	+
Olansky [2011]	?	?	+	+	+	+	+
PIONEER-3	+	+	+	+	+	+	+
PIONEER-6	?	?	+	+	?	+	+
PIONEER-8	+	+	+	+	+	+	+
Perez [2010]	+	+	+	+	+	+	+
Pinget [2013]	+	+	+	+	+	+	+
Pratley [2018]	?	?	+	?	+	+	+
Perkovic [2019]	+	+	+	+	+	+	+
Pfutzner [2011]	+	+	+	+	+	+	+
REWIND	+	+	+	+	?	+	+
Ridderstrale [2018]	+	+	+	+	+	+	+
Roden [2015]	+	+	+	+	+	+	+
Rosenstock [2013]	?	?	+	+	+	+	+
Rosenstock [2014]	+	+	+	+	+	+	+
Rosenstock [2015]	+	+	+	+	+	+	+
Rosenstock [2019]	+	+	+	+	+	+	+
SCORED	+	+	+	+	+	+	+
SAVOR-TIMI 53	+	+	+	+	?	+	+
SCALE	+	+	+	+	+	+	+
SUSTAIN-1	+	+	+	+	+	+	+
SUSTAIN-6	?	+	+	+	?	+	+
SUSTAIN-8	+	+	+	+	+	+	+
Schernthaner [2013]	+	+	+	+	+	+	+
Schernthaner [2015]	+	?	+	+	?	?	+
Seck [2010]	?	+	+	+	+	+	+
Scott [2018]	+	+	+	+	+	+	+
Schweizer [2009]	+	+	+	+	+	+	+
Seino [2010]	+	+	+	+	+	+	+
Seino [2016]	+	+	+	+	+	+	+
Soloist-WHF	+	+	+	+	+	+	+
Strojek [2014]	+	+	+	+	+	+	+
TECOS	+	+	+	+	+	+	+
Tolman [2009]	+	+	+	+	+	+	+
VADT	+	+	+	+	+	+	+
Wainstein [2011]	?	?	+	+	+	+	+
Wilding [2012]	+	+	+	+	+	+	+
Wilding [2013]	+	+	+	+	+	+	+
Yale [2014]	?	+	+	+	+	+	+
Yang [2018]	+	+	+	+	+	+	+

Key
- \+ Low risk of bias
- − High risk of bias
- ? Unclear risk of bias

Table S3. Egger's Regression Tests for Funnel Plot Asymmetry

		t	z	p-value
MACE				
	weighted regression with multiplicative dispersion	-1.3508		0.1787
	mixed-effects meta-regression model		-1.0985	0.2720
Non-fatal MI				
	weighted regression with multiplicative dispersion	-0.9182		0.3598
	mixed-effects meta-regression model		0.0574	0.9542
All-cause death				
	weighted regression with multiplicative dispersion	0.5530		0.5811
	mixed-effects meta-regression model		0.5723	0.5671

Figure S1. Evaluation of publication bias in funnel plots for (a) MACE; (b) All-cause deaths; (c) Non-fatal myocardial infarction

(a)

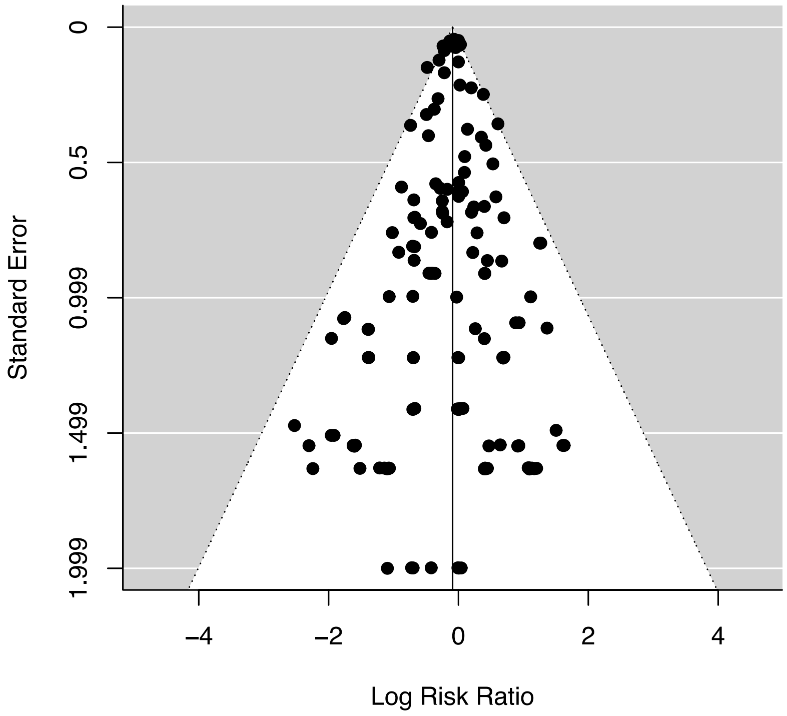

(b)

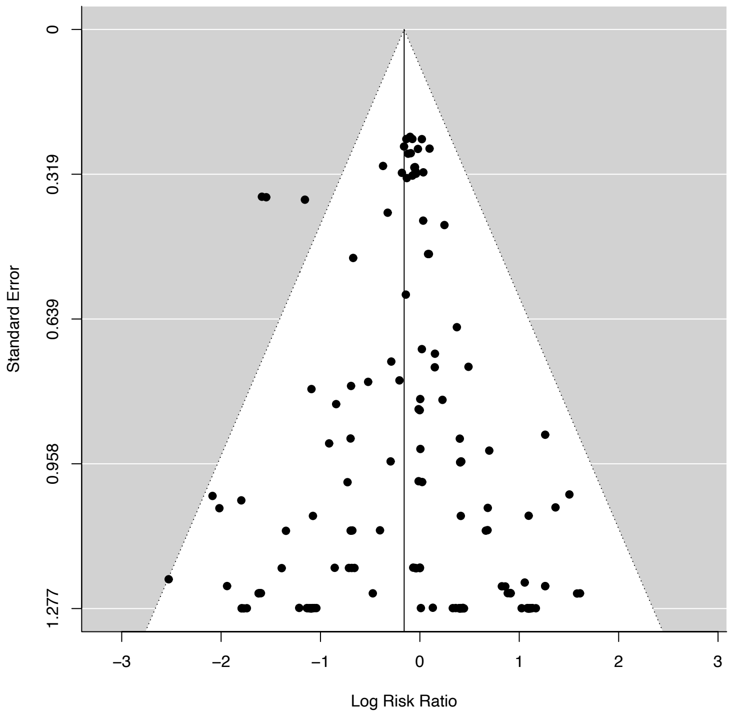

(c)

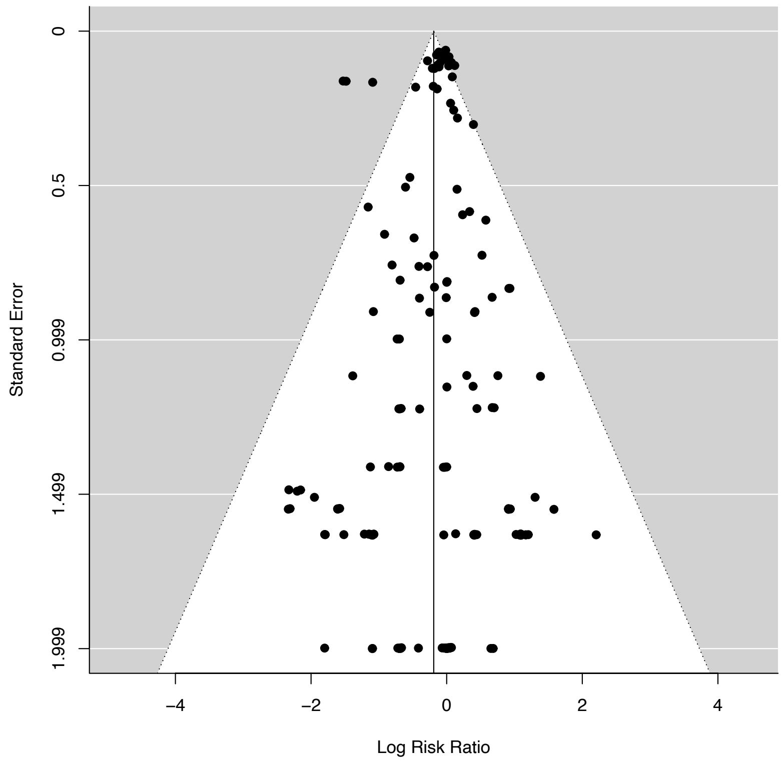

Figure S2. Sensitivity analysis with exclusion of one study with the largest exposure (sample size * follow-up time) per drug in the active arm. Forest plot comparing antidiabetic therapies for the occurrence of major cardiovascular adverse events (MACE) in an additive effects network meta-analysis with a random-effects model.

(a) Sensitivity analysis 1

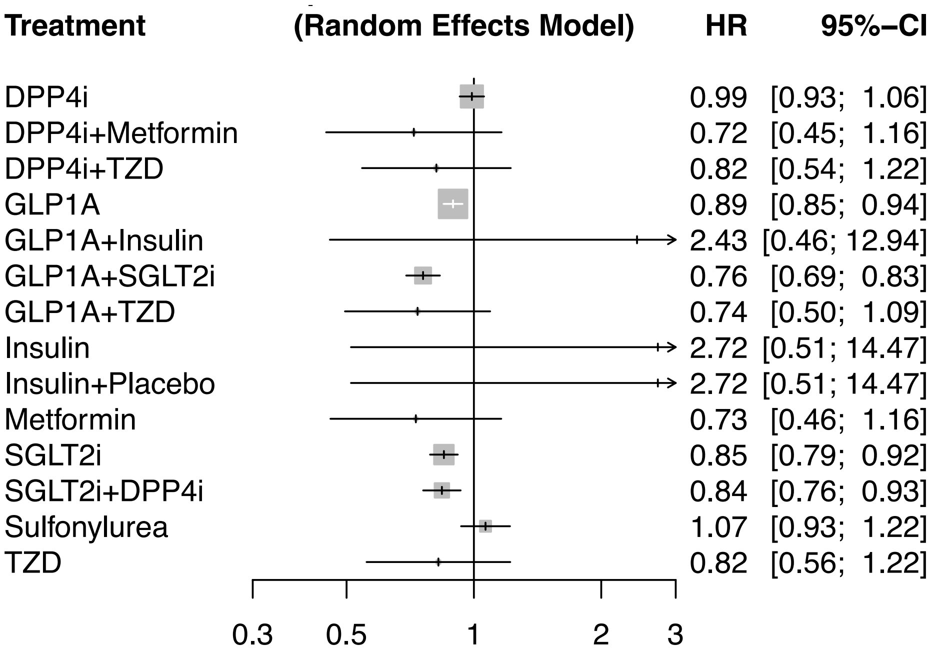

The excluded trials were: TECOS, REWIND, DECLARE, Dormandy (2005). The reference group was considered as placebo. Number of pairwise comparisons was 131 (118 studies with >100 individuals per arm), number of treatments (n = 19), number of designs (n=37) with 7 active components. Heterogeneity / inconsistency analysis showed $tau^2 = 0$; tau = 0; $I^2 = 0\%$ [0.0%; 0.0%]; Q = 98.58 (p=0.9890). Among TZDs (thiazolidinediones) only pioglitazone was included.

(b) Sensitivity analysis 2

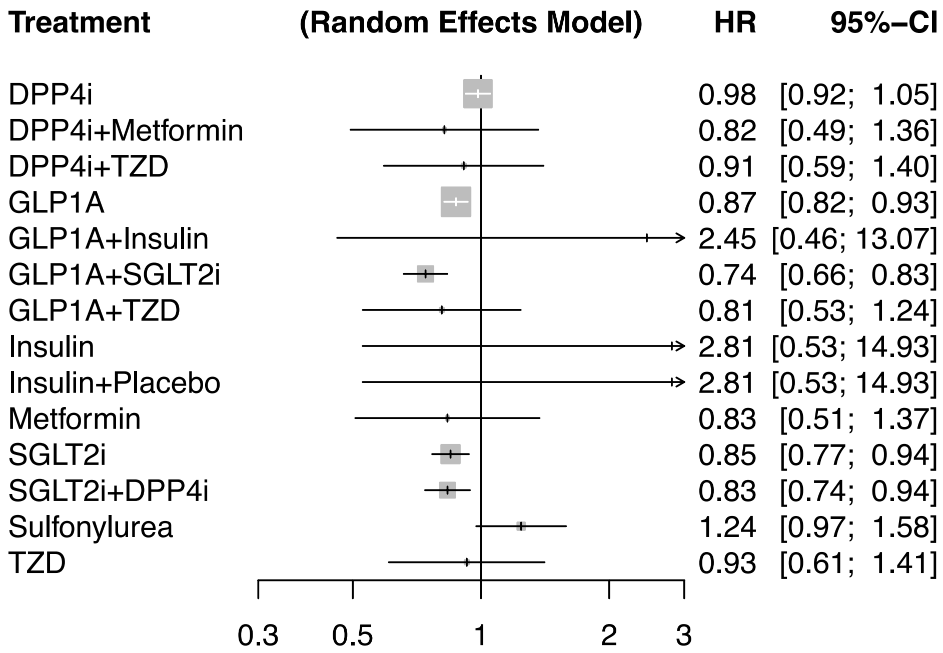

The excluded trials were: TECOS, CAROLINA, REWIND, EXCEL, DECLARE, CANVAS, Dormandy (2005). Number of pairwise comparisons was 128 (115 studies with >100 individuals per arm), number of treatments (n = 19), number of designs (n=37) with 7 active components. Heterogeneity / inconsistency analysis showed $tau^2 = 0$; tau = 0; $I^2 = 0\%$ [0.0%; 0.0%]; Q = 84.01 (p=0.9912). Among TZDs (thiazolidinediones) only pioglitazone was included.

Figure S3. Sensitivity analysis considering (a) all-cause death or (b) non-fatal MI as outcomes

(a)

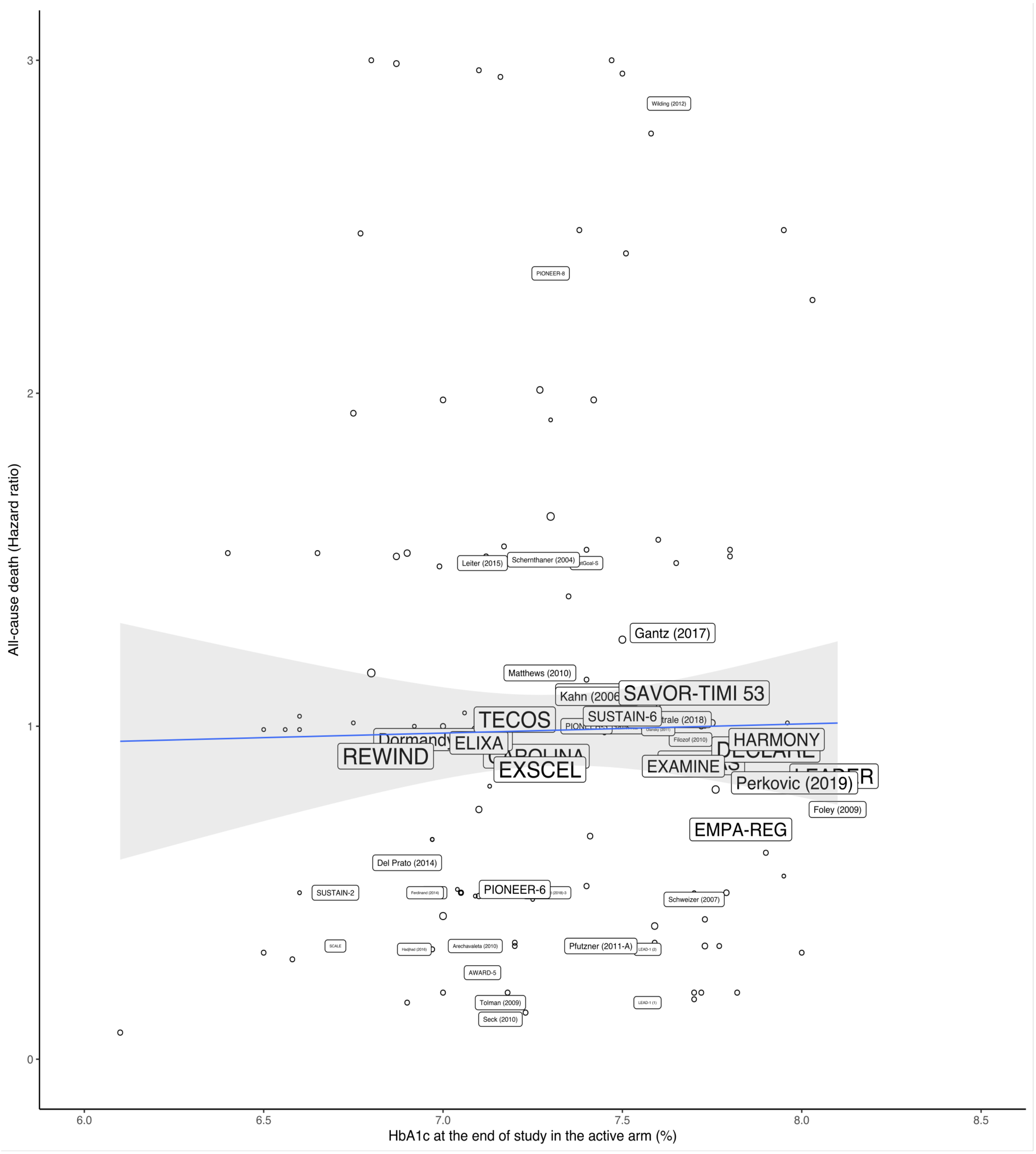

(b)

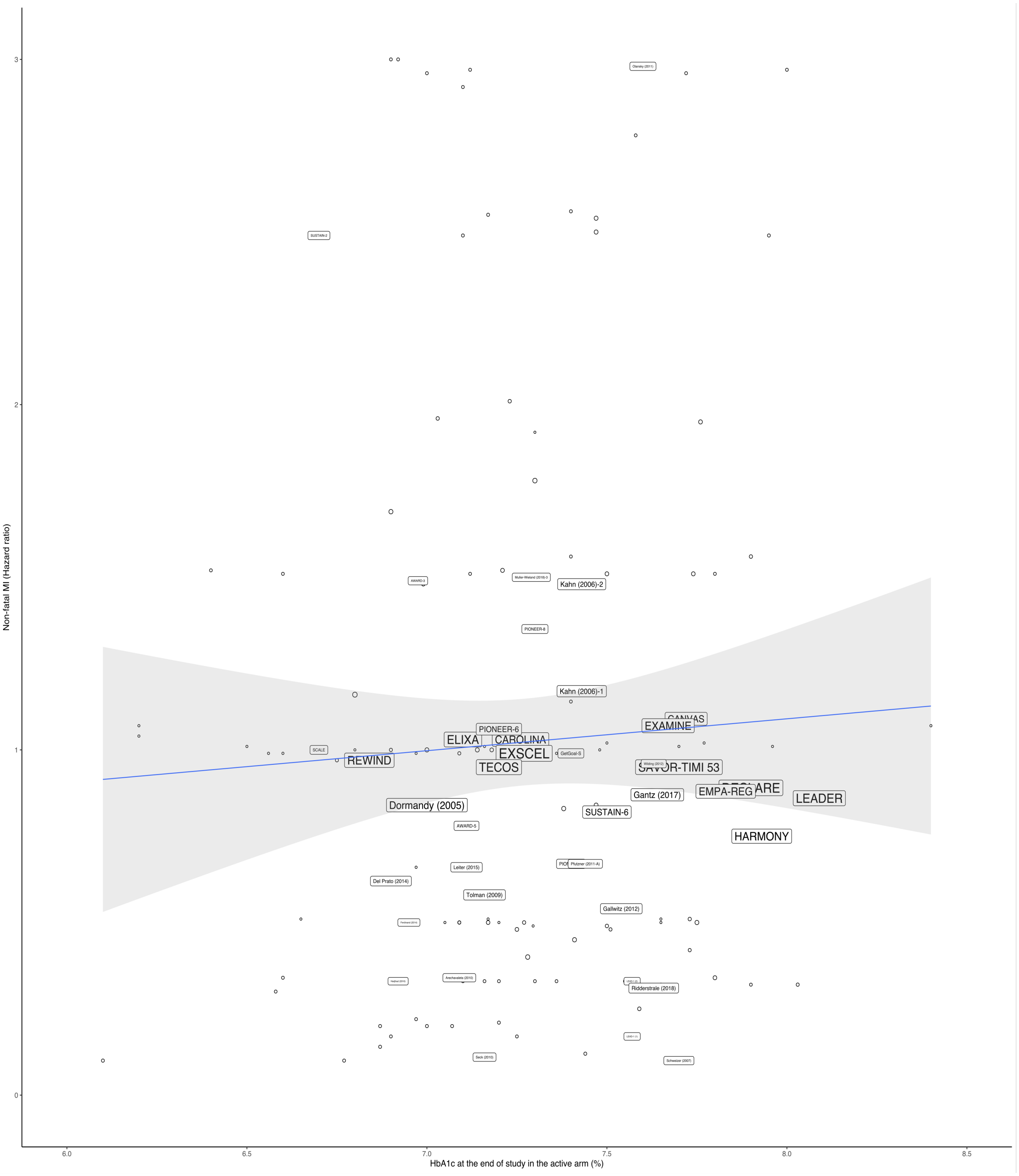
Non-fatal MI (Hazard ratio)
HbA1c at the end of study in the active arm (%)
0
1
2
3
6.0
6.5
7.0
7.5
8.0
8.5
SUSTAIN-2
AWARD-3
Kahn (2006)-2
PIONEER-8
Kahn (2006)-1
PIONEER-6
EXAMINE
ELIXA
CAROLINA
EXSCEL
GetGoal-S
SCALE
REWIND
TECOS
Gantz (2017)
EMPA-REG
LEADER
Dormandy (2005)
SUSTAIN-6
AWARD-5
HARMONY
Leiter (2015)
Del Prato (2014)
Tolman (2009)
Gallwitz (2012)
Arechavaleta (2010)
Ridderstrale (2018)
Seck (2010)
Schweizer (2007)

Figure S4. Relationship between weight change and the HR for MACE

Name	Active	Control	HR [95% CI]
AWARD−3	2/539	0/268	2.49 [0.12, 51.70]
AWARD−9	2/150	0/150	5.00 [0.24, 103.28]
Bailey (2013)	6/409	4/137	0.50 [0.14, 1.75]
Bailey (2015)	2/199	0/75	1.90 [0.09, 39.12]
Barnett (2014)	1/187	1/187	1.00 [0.06, 15.87]
Bode (2015)	9/477	3/237	1.49 [0.41, 5.45]
Buse (2011)	0/137	1/122	0.30 [0.01, 7.23]
CONFIDENCE (1)	1/110	1/114	1.04 [0.07, 16.36]
CONFIDENCE (2)	1/110	1/118	1.07 [0.07, 16.94]
Cefalu (2015)	6/455	3/459	2.02 [0.51, 8.02]
Chen (2018)	1/478	0/242	1.52 [0.06, 37.22]
DURATION−2 (1)	0/160	1/166	0.35 [0.01, 8.43]
DURATION−2 (2)	0/160	3/165	0.15 [0.01, 2.83]
DURATION−4 (1)	0/248	1/246	0.33 [0.01, 8.08]
DURATION−4 (2)	0/248	1/163	0.22 [0.01, 5.36]
DURATION−4 (3)	0/248	0/163	0.66 [0.01, 33.03]
DURATION−7	0/231	1/230	0.33 [0.01, 8.11]
DURATION−8 (1)	5/227	4/230	1.27 [0.34, 4.66]
DURATION−8 (2)	2/228	4/230	0.50 [0.09, 2.73]
DURATION−8 (3)	2/228	5/227	0.40 [0.08, 2.03]
Dagogo−Jack (2017)	0/309	0/153	0.50 [0.01, 24.92]
Davies (2021)	11/807	3/403	1.83 [0.51, 6.53]
Del Prato (2015)	1/400	4/401	0.25 [0.03, 2.23]
Ferdinand (2014)	1/505	1/250	0.50 [0.03, 7.88]
Gallwitz (2012)	11/776	23/775	0.48 [0.23, 0.97]
GetGoal−L	3/328	3/167	0.51 [0.10, 2.50]
GetGoal−M−Asia	2/196	1/194	1.98 [0.18, 21.65]
GetGoal−O	0/176	3/174	0.14 [0.01, 2.71]
GetGoal−S	8/574	4/285	0.99 [0.30, 3.27]
Goke (2013)	3/428	6/430	0.50 [0.13, 2.00]
HARMONY	338/4731	428/4732	0.79 [0.69, 0.91]
HARMONY−3 (1)	7/302	2/307	3.56 [0.75, 16.99]
HARMONY−3 (2)	7/302	2/302	3.50 [0.73, 16.71]
HARMONY−3 (3)	7/302	3/101	0.78 [0.21, 2.96]
HARMONY−5 (1)	1/271	6/277	0.17 [0.02, 1.41]
HARMONY−5 (2)	1/271	3/115	0.14 [0.01, 1.35]
Hadjhad (2016)	0/995	0/332	0.33 [0.01, 16.82]
Haering (2015)	4/441	4/225	0.51 [0.13, 2.02]
Hollander (2019)	17/870	5/435	1.70 [0.63, 4.58]
IDegLira	1/199	2/199	0.50 [0.05, 5.47]
Ji (2021)	1/578	1/290	0.50 [0.03, 7.99]
Kovacs (2015)	4/333	3/165	0.66 [0.15, 2.92]
LIRA−RENAL	4/140	1/137	3.91 [0.44, 34.58]
LIRA−SWITCH	0/202	1/204	0.34 [0.01, 8.21]
Leiter (2014)	6/480	6/482	1.00 [0.33, 3.09]
Leiter (2015)	11/968	5/482	1.10 [0.38, 3.14]
Lewin (2015)	1/265	2/133	0.25 [0.02, 2.74]
Lewin (2015)−1	2/269	2/133	0.49 [0.07, 3.47]
Lewin (2015)−2	1/265	2/133	0.25 [0.02, 2.74]
Mathieu (2016)	1/160	0/160	3.00 [0.12, 73.09]
Merker (2015)	3/430	4/207	0.36 [0.08, 1.60]
Muller−Wieland (2018)−1	2/314	1/313	1.99 [0.18, 21.87]
Muller−Wieland (2018)−2	1/312	1/313	1.00 [0.06, 15.97]
Muller−Wieland (2018)−3	4/626	0/313	4.51 [0.24, 83.45]
Olansky (2011)	1/625	0/621	2.98 [0.12, 73.03]
PIONEER−3	16/1396	3/467	1.78 [0.52, 6.10]
PIONEER−6	61/1591	76/1592	0.80 [0.58, 1.12]
PIONEER−8	9/547	4/184	0.76 [0.24, 2.43]
Pratley (2018)	1/498	0/247	1.49 [0.06, 36.47]
REWIND	594/4949	663/4952	0.90 [0.81, 0.99]
Roden (2015)−1	5/448	2/223	1.24 [0.24, 6.36]
Roden (2015)−2	5/448	1/228	2.54 [0.30, 21.65]
Rosenstock (2014)	2/375	1/188	1.00 [0.09, 10.99]
Rosenstock (2015)−1	7/324	3/170	1.22 [0.32, 4.67]
Rosenstock (2019)−1	0/290	1/289	0.33 [0.01, 8.12]
Rosenstock (2019)−2	0/290	0/291	1.00 [0.02, 50.40]
SCALE	5/634	3/212	0.56 [0.13, 2.31]
SCORED	400/5292	530/5292	0.75 [0.67, 0.85]
SUSTAIN−1	1/258	0/129	1.51 [0.06, 36.71]
SUSTAIN−6	108/1648	146/1649	0.74 [0.58, 0.94]
SUSTAIN−8	1/394	1/394	1.00 [0.06, 15.93]
Schernthaner (2013)	3/377	2/378	1.50 [0.25, 8.95]
Seck (2010)	1/588	4/584	0.25 [0.03, 2.21]
Strojek (2014)	4/447	1/145	1.30 [0.15, 11.52]
Wilding (2012)	2/607	0/193	1.60 [0.08, 33.09]
Wilding (2013)	1/313	2/156	0.25 [0.02, 2.73]
Yale (2014)	5/179	3/90	0.84 [0.20, 3.43]
Yang (2018)	0/139	1/133	0.32 [0.01, 7.76]
Weight loss superior to 1kg	1736/46411	2013/36458	0.82 [0.77, 0.87]
Arjona (2013)	5/211	5/212	1.00 [0.30, 3.42]
Bosi (2007)	1/362	0/182	1.51 [0.06, 36.94]
DeFronzo (2009)	0/564	1/179	0.11 [0.00, 2.60]
Del Prato (2014)	14/1765	11/874	0.63 [0.29, 1.38]
Gantz (2017)	114/2100	114/2102	1.00 [0.78, 1.29]
GetGoal−L−Asia	2/154	0/157	5.10 [0.25, 105.31]
HARMONY−1	2/150	1/151	2.01 [0.18, 21.97]
HARMONY−8	5/249	7/246	0.71 [0.23, 2.19]
Handelsman (2017)	3/376	2/375	1.50 [0.25, 8.90]
Henry (2013)−1	2/186	2/565	3.04 [0.43, 21.41]
Henry (2013)−2	2/186	4/581	1.56 [0.29, 8.46]
Kohan (2014)	5/168	6/84	0.42 [0.13, 1.33]
Matthews (2010)	15/1562	13/1556	1.15 [0.55, 2.41]
Moses (2015)	1/210	0/212	3.03 [0.12, 73.92]
Pfutzner (2011−A)	7/976	3/328	0.78 [0.20, 3.01]
Rosenstock (2013)	0/222	2/219	0.20 [0.01, 4.09]
Schernthaner (2015)	4/360	3/360	1.33 [0.30, 5.91]
Schweizer (2009)	1/169	1/166	0.98 [0.06, 15.57]
Seino (2010)	3/268	3/132	0.49 [0.10, 2.41]
Yang (2015)−2	0/299	0/145	0.49 [0.01, 24.40]
Neutral weight change	186/10537	178/8826	0.96 [0.78, 1.17]
AWARD−5	6/606	4/315	0.78 [0.22, 2.74]
Abdul−ghani (2017)	0/123	5/108	0.08 [0.00, 1.43]
Arechavaleta (2010)	0/516	2/519	0.20 [0.01, 4.18]
Barnett (2013)	2/304	1/151	0.99 [0.09, 10.87]
Bolli (2009)	2/295	3/281	0.64 [0.11, 3.77]
Bosi (2011)	2/404	3/399	0.66 [0.11, 3.92]
Chacra (2011)	8/502	4/267	1.06 [0.32, 3.50]
Charpentier (2009)	1/142	0/147	3.10 [0.13, 75.59]
DUAL IV	2/289	0/146	2.53 [0.12, 52.45]
EXAMINE	305/2701	316/2679	0.96 [0.83, 1.11]
Ferrannini (2009)	22/1393	12/1396	1.84 [0.91, 3.70]
GET−GOAL	3/322	1/160	1.49 [0.16, 14.22]
GetGoal−Duo−1	1/223	2/223	0.50 [0.05, 5.47]
Giles (2010)	0/151	2/149	0.20 [0.01, 4.08]
Goke (2008)	1/305	0/158	1.56 [0.06, 38.05]
Henry (2013)−3	4/581	2/565	1.94 [0.36, 10.58]
Hollander (2011)	5/381	1/184	2.41 [0.28, 20.52]
Jain (2006)	2/251	3/251	0.67 [0.11, 3.96]
Mazzone (2006)	0/230	2/228	0.20 [0.01, 4.11]
Nauck (2016)−1	2/392	3/409	0.70 [0.12, 4.14]
Nauck (2016)−2	2/392	2/135	0.34 [0.05, 2.42]
Nissen (2008)	5/270	6/273	0.84 [0.26, 2.73]
Pinget (2013)	0/323	2/161	0.10 [0.00, 2.07]
Seino (2016)	0/127	2/130	0.20 [0.01, 4.22]
Tolman (2009)	18/1051	26/1046	0.69 [0.38, 1.25]
Wainstein (2011)	0/256	1/261	0.34 [0.01, 8.30]
Weight gain	393/12530	405/10741	0.94 [0.82, 1.07]

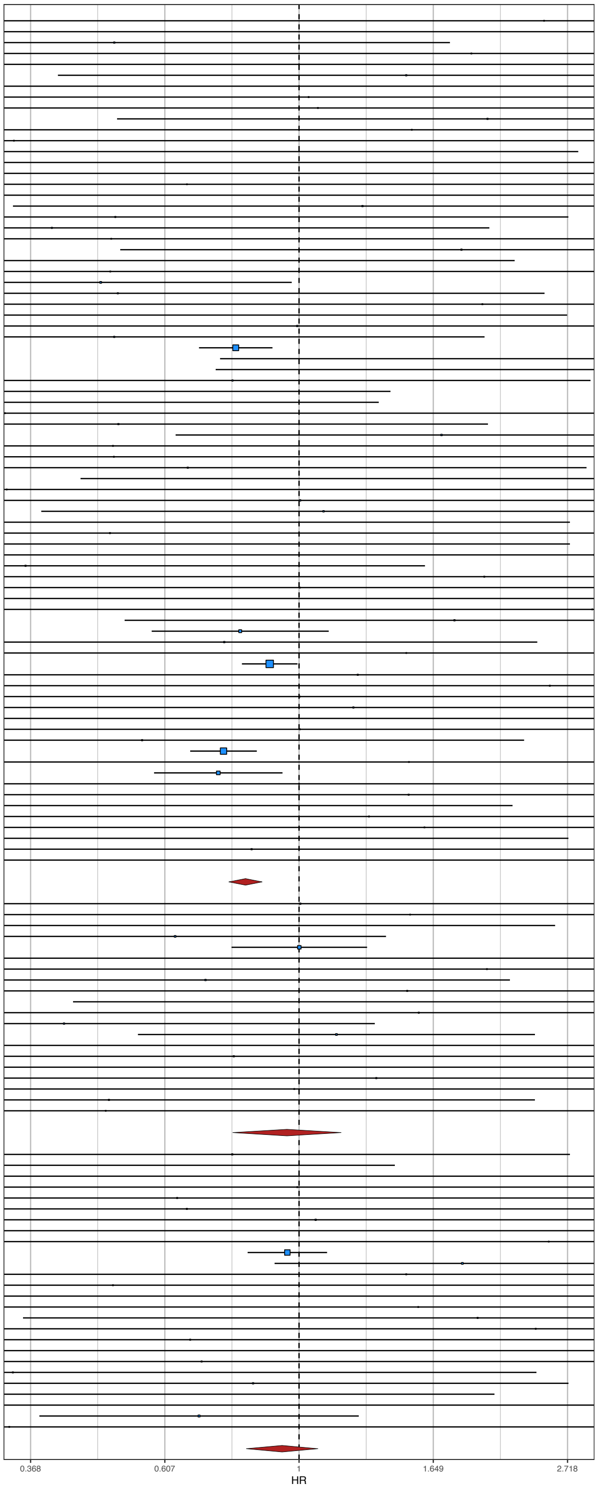

Figure S5.

Name	Active	Control	HR [95% CI]
AWARD−3	2/539	0/268	2.49 [0.12, 51.70]
AWARD−5	6/606	4/315	0.78 [0.22, 2.74]
AWARD−9	2/150	0/150	5.00 [0.24, 103.28]
Abdul−ghani (2017)	0/123	5/108	0.08 [0.00, 1.43]
Arechavaleta (2010)	0/516	2/519	0.20 [0.01, 4.18]
Arjona (2013)	5/211	5/212	1.00 [0.30, 3.42]
Bailey (2013)	6/409	4/137	0.50 [0.14, 1.75]
Bailey (2015)	2/199	0/75	1.90 [0.09, 39.12]
Barnett (2013)	2/304	1/151	0.99 [0.09, 10.87]
Barnett (2014)	1/187	1/187	1.00 [0.06, 15.87]
Bode (2015)	9/477	3/237	1.49 [0.41, 5.45]
Bolli (2009)	2/295	3/281	0.64 [0.11, 3.77]
Bosi (2007)	1/362	0/182	1.51 [0.06, 36.94]
Buse (2011)	0/137	1/122	0.30 [0.01, 7.23]
CANVAS	540/5795	471/4347	0.86 [0.76, 0.97]
CARMELINA	434/3494	420/3485	1.03 [0.91, 1.17]
CAROLINA	356/3023	362/3010	0.98 [0.85, 1.12]
Chen (2018)	1/478	0/242	1.52 [0.06, 37.22]
DECLARE	756/8582	803/8578	0.94 [0.86, 1.03]
DURATION−8 (1)	5/227	4/230	1.27 [0.34, 4.66]
DURATION−8 (2)	2/228	4/230	0.50 [0.09, 2.73]
DURATION−8 (3)	2/228	5/227	0.40 [0.08, 2.03]
Dagogo−Jack (2017)	0/309	0/153	0.50 [0.01, 24.92]
Del Prato (2014)	14/1765	11/874	0.63 [0.29, 1.38]
Del Prato (2015)	1/400	4/401	0.25 [0.03, 2.23]
ELIXA	397/3034	389/3034	1.02 [0.90, 1.16]
Ferdinand (2014)	1/505	1/250	0.50 [0.03, 7.88]
GET−GOAL	3/322	1/160	1.49 [0.16, 14.22]
Gallwitz (2012)	11/776	23/775	0.48 [0.23, 0.97]
GetGoal−Duo−1	1/223	2/223	0.50 [0.05, 5.47]
GetGoal−M−Asia	2/196	1/194	1.98 [0.18, 21.65]
GetGoal−S	8/574	4/285	0.99 [0.30, 3.27]
Goke (2008)	1/305	0/158	1.56 [0.06, 38.05]
Goke (2013)	3/428	6/430	0.50 [0.13, 2.00]
HARMONY	338/4731	428/4732	0.79 [0.69, 0.91]
HARMONY−3 (1)	7/302	2/307	3.56 [0.75, 16.99]
HARMONY−3 (2)	7/302	2/302	3.50 [0.73, 16.71]
HARMONY−3 (3)	7/302	3/101	0.78 [0.21, 2.96]
HARMONY−5 (1)	1/271	6/277	0.17 [0.02, 1.41]
HARMONY−5 (2)	1/271	3/115	0.14 [0.01, 1.35]
HARMONY−8	5/249	7/246	0.71 [0.23, 2.19]
Hadjhad (2016)	0/995	0/332	0.33 [0.01, 16.82]
Handelsman (2017)	3/376	2/375	1.50 [0.25, 8.90]
Henry (2013)−3	4/581	2/565	1.94 [0.36, 10.58]
Hollander (2011)	5/381	1/184	2.41 [0.28, 20.52]
Hollander (2019)	17/870	5/435	1.70 [0.63, 4.58]
Kawamori (2018)	1/182	1/93	0.51 [0.03, 8.08]
Kohan (2014)	5/168	6/84	0.42 [0.13, 1.33]
Kovacs (2015)	4/333	3/165	0.66 [0.15, 2.92]
LEADER	608/4668	694/4672	0.88 [0.79, 0.97]
LIRA−SWITCH	0/202	1/204	0.34 [0.01, 8.21]
Leiter (2015)	11/968	5/482	1.10 [0.38, 3.14]
Mathieu (2016)	1/160	0/160	3.00 [0.12, 73.09]
Matthews (2010)	15/1562	13/1556	1.15 [0.55, 2.41]
Merker (2015)	3/430	4/207	0.36 [0.08, 1.60]
Muller−Wieland (2018)−1	2/314	1/313	1.99 [0.18, 21.87]
Muller−Wieland (2018)−2	1/312	1/313	1.00 [0.06, 15.97]
Muller−Wieland (2018)−3	4/626	0/313	4.51 [0.24, 83.45]
Nauck (2016)−2	2/392	2/135	0.34 [0.05, 2.42]
Olansky (2011)	1/625	0/621	2.98 [0.12, 73.03]
PIONEER−3	16/1396	3/467	1.78 [0.52, 6.10]
REWIND	594/4949	663/4952	0.90 [0.81, 0.99]
Roden (2015)−1	5/448	2/223	1.24 [0.24, 6.36]
Roden (2015)−2	5/448	1/228	2.54 [0.30, 21.65]
Rosenstock (2013)	0/222	2/219	0.20 [0.01, 4.09]
Rosenstock (2014)	2/375	1/188	1.00 [0.09, 10.99]
Rosenstock (2019)−1	0/290	1/289	0.33 [0.01, 8.12]
SCORED	400/5292	530/5292	0.75 [0.67, 0.85]
SUSTAIN−6	108/1648	146/1649	0.74 [0.58, 0.94]
Schernthaner (2015)	4/360	3/360	1.33 [0.30, 5.91]
Scott (2018)	0/306	1/307	0.33 [0.01, 8.18]
Seck (2010)	1/588	4/584	0.25 [0.03, 2.21]
Seino (2010)	3/268	3/132	0.49 [0.10, 2.41]
Strojek (2014)	4/447	1/145	1.30 [0.15, 11.52]
Wainstein (2011)	0/256	1/261	0.34 [0.01, 8.30]
Wilding (2012)	2/607	0/193	1.60 [0.08, 33.09]
Wilding (2013)	1/313	2/156	0.25 [0.02, 2.73]
Yang (2015)−2	0/299	0/145	0.49 [0.01, 24.40]
Yang (2018)	0/139	1/133	0.32 [0.01, 7.76]
Severe hypoglycemia more frequent in control arm	4774/74631	5097/63442	0.89 [0.86, 0.93]
Bosi (2011)	2/404	3/399	0.66 [0.11, 3.92]
DUAL IV	2/289	0/146	2.53 [0.12, 52.45]
EXAMINE	305/2701	316/2679	0.96 [0.83, 1.11]
EXSCEL	839/7356	905/7396	0.93 [0.85, 1.02]
Ferrannini (2009)	22/1393	12/1396	1.84 [0.91, 3.70]
Gantz (2017)	114/2100	114/2102	1.00 [0.78, 1.29]
GetGoal−L	3/328	3/167	0.51 [0.10, 2.50]
HARMONY−1	2/150	1/151	2.01 [0.18, 21.97]
Henry (2013)−1	2/186	2/565	3.04 [0.43, 21.41]
Henry (2013)−2	2/186	4/581	1.56 [0.29, 8.46]
Lewin (2015)	1/265	2/133	0.25 [0.02, 2.74]
Nauck (2016)−1	2/392	3/409	0.70 [0.12, 4.14]
PIONEER−6	61/1591	76/1592	0.80 [0.58, 1.12]
PIONEER−8	9/547	4/184	0.76 [0.24, 2.43]
Pratley (2018)	1/498	0/247	1.49 [0.06, 36.47]
Rosenstock (2019)−2	0/290	0/291	1.00 [0.02, 50.40]
SAVOR−TIMI 53	613/8280	609/8212	1.00 [0.90, 1.11]
SCALE	5/634	3/212	0.56 [0.13, 2.31]
SUSTAIN−8	1/394	1/394	1.00 [0.06, 15.93]
Schernthaner (2013)	3/377	2/378	1.50 [0.25, 8.95]
TECOS	745/7332	746/7339	1.00 [0.91, 1.10]
Yale (2014)	5/179	3/90	0.84 [0.20, 3.43]
Severe hypoglycemia more frequent in active arm	3316/39085	3551/38310	0.97 [0.92, 1.02]

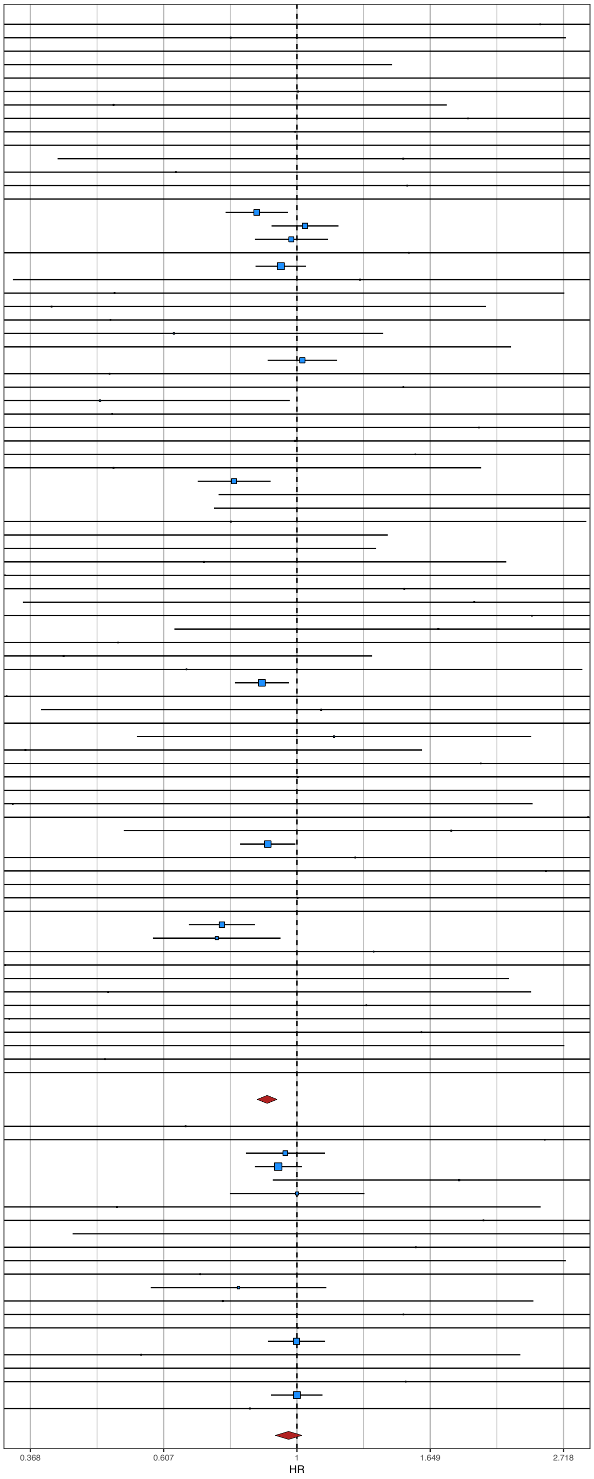

REFERENCES

1. Gerstein HC, Miller ME, Byington RP, Goff DC, Bigger JT, Buse JB, et al. Effects of intensive glucose lowering in type 2 diabetes. N Engl J Med. 2008;358(24):2545-59.
2. Patel A, MacMahon S, Chalmers J, Neal B, Billot L, Woodward M, et al. Intensive blood glucose control and vascular outcomes in patients with type 2 diabetes. N Engl J Med. 2008;358(24):2560-72.
3. Umpierrez G, Tofé Povedano S, Pérez Manghi F, Shurzinske L, Pechtner V. Efficacy and safety of dulaglutide monotherapy versus metformin in type 2 diabetes in a randomized controlled trial (AWARD-3). Diabetes Care. 2014;37(8):2168-76.
4. Weinstock RS, Guerci B, Umpierrez G, Nauck MA, Skrivanek Z, Milicevic Z. Safety and efficacy of once-weekly dulaglutide versus sitagliptin after 2 years in metformin-treated patients with type 2 diabetes (AWARD-5): a randomized, phase III study. Diabetes Obes Metab. 2015;17(9):849-58.
5. Pozzilli P, Norwood P, Jódar E, Davies MJ, Ivanyi T, Jiang H, et al. Placebo-controlled, randomized trial of the addition of once-weekly glucagon-like peptide-1 receptor agonist dulaglutide to titrated daily insulin glargine in patients with type 2 diabetes (AWARD-9). Diabetes Obes Metab. 2017;19(7):1024-31.
6. Abdul-Ghani M, Migahid O, Megahed A, Adams J, Triplitt C, DeFronzo RA, et al. Combination Therapy With Exenatide Plus Pioglitazone Versus Basal/Bolus Insulin in Patients With Poorly Controlled Type 2 Diabetes on Sulfonylurea Plus Metformin: The Qatar Study. Diabetes Care. 2017;40(3):325-31.
7. Arechavaleta R, Seck T, Chen Y, Krobot KJ, O'Neill EA, Duran L, et al. Efficacy and safety of treatment with sitagliptin or glimepiride in patients with type 2 diabetes inadequately controlled on metformin monotherapy: a randomized, double-blind, non-inferiority trial. Diabetes Obes Metab. 2011;13(2):160-8.
8. Arjona Ferreira JC, Marre M, Barzilai N, Guo H, Golm GT, Sisk CM, et al. Efficacy and safety of sitagliptin versus glipizide in patients with type 2 diabetes and moderate-to-severe chronic renal insufficiency. Diabetes Care. 2013;36(5):1067-73.
9. Bailey CJ, Gross JL, Hennicken D, Iqbal N, Mansfield TA, List JF. Dapagliflozin add-on to metformin in type 2 diabetes inadequately controlled with metformin: a randomized, double-blind, placebo-controlled 102-week trial. BMC Med. 2013;11:43.
10. Bailey CJ, Morales Villegas EC, Woo V, Tang W, Ptaszynska A, List JF. Efficacy and safety of dapagliflozin monotherapy in people with Type 2 diabetes: a randomized double-blind placebo-controlled 102-week trial. Diabet Med. 2015;32(4):531-41.
11. Barnett AH, Charbonnel B, Li J, Donovan M, Fleming D, Iqbal N. Saxagliptin add-on therapy to insulin with or without metformin for type 2 diabetes mellitus: 52-week safety and efficacy. Clin Drug Investig. 2013;33(10):707-17.
12. Barnett AH, Mithal A, Manassie J, Jones R, Rattunde H, Woerle HJ, et al. Efficacy and safety of empagliflozin added to existing antidiabetes treatment in patients with type 2 diabetes and chronic kidney disease: a randomised, double-blind, placebo-controlled trial. Lancet Diabetes Endocrinol. 2014;2(5):369-84.
13. Bode B, Stenlöf K, Harris S, Sullivan D, Fung A, Usiskin K, et al. Long-term efficacy and safety of canagliflozin over 104 weeks in patients aged 55-80 years with type 2 diabetes. Diabetes Obes Metab. 2015;17(3):294-303.
14. Bolli G, Dotta F, Colin L, Minic B, Goodman M. Comparison of vildagliptin and pioglitazone in patients with type 2 diabetes inadequately controlled with metformin. Diabetes Obes Metab. 2009;11(6):589-95.

15. Bosi E, Camisasca RP, Collober C, Rochotte E, Garber AJ. Effects of vildagliptin on glucose control over 24 weeks in patients with type 2 diabetes inadequately controlled with metformin. Diabetes Care. 2007;30(4):890-5.
16. Bosi E, Ellis GC, Wilson CA, Fleck PR. Alogliptin as a third oral antidiabetic drug in patients with type 2 diabetes and inadequate glycaemic control on metformin and pioglitazone: a 52-week, randomized, double-blind, active-controlled, parallel-group study. Diabetes Obes Metab. 2011;13(12):1088-96.
17. Buse JB, Bergenstal RM, Glass LC, Heilmann CR, Lewis MS, Kwan AY, et al. Use of twice-daily exenatide in Basal insulin-treated patients with type 2 diabetes: a randomized, controlled trial. Ann Intern Med. 2011;154(2):103-12.
18. Neal B, Perkovic V, Mahaffey KW, de Zeeuw D, Fulcher G, Erondu N, et al. Canagliflozin and Cardiovascular and Renal Events in Type 2 Diabetes. N Engl J Med. 2017;377(7):644-57.
19. Rosenstock J, Perkovic V, Johansen OE, Cooper ME, Kahn SE, Marx N, et al. Effect of Linagliptin vs Placebo on Major Cardiovascular Events in Adults With Type 2 Diabetes and High Cardiovascular and Renal Risk: The CARMELINA Randomized Clinical Trial. JAMA. 2019;321(1):69-79.
20. Rosenstock J, Kahn SE, Johansen OE, Zinman B, Espeland MA, Woerle HJ, et al. Effect of Linagliptin vs Glimepiride on Major Adverse Cardiovascular Outcomes in Patients With Type 2 Diabetes: The CAROLINA Randomized Clinical Trial. JAMA. 2019.
21. Xu W, Bi Y, Sun Z, Li J, Guo L, Yang T, et al. Comparison of the effects on glycaemic control and β-cell function in newly diagnosed type 2 diabetes patients of treatment with exenatide, insulin or pioglitazone: a multicentre randomized parallel-group trial (the CONFIDENCE study). J Intern Med. 2015;277(1):137-50.
22. Cefalu WT, Leiter LA, de Bruin TW, Gause-Nilsson I, Sugg J, Parikh SJ. Dapagliflozin's Effects on Glycemia and Cardiovascular Risk Factors in High-Risk Patients With Type 2 Diabetes: A 24-Week, Multicenter, Randomized, Double-Blind, Placebo-Controlled Study With a 28-Week Extension. Diabetes Care. 2015;38(7):1218-27.
23. Chacra AR, Tan GH, Ravichandran S, List J, Chen R, Investigators C. Safety and efficacy of saxagliptin in combination with submaximal sulphonylurea versus up-titrated sulphonylurea over 76 weeks. Diab Vasc Dis Res. 2011;8(2):150-9.
24. Charpentier G, Halimi S, Investigators F-P-S. Earlier triple therapy with pioglitazone in patients with type 2 diabetes. Diabetes Obes Metab. 2009;11(9):844-54.
25. Chen YH, Huang CN, Cho YM, Li P, Gu L, Wang F, et al. Efficacy and safety of dulaglutide monotherapy compared with glimepiride in East-Asian patients with type 2 diabetes in a multicentre, double-blind, randomized, parallel-arm, active comparator, phase III trial. Diabetes Obes Metab. 2018;20(9):2121-30.
26. Davies M, Færch L, Jeppesen OK, et al. Semaglutide 2·4 mg once a week in adults with overweight or obesity, and type 2 diabetes (STEP 2): a randomised, double-blind, double-dummy, placebo-controlled, phase 3 trial. Lancet. 2021;397(10278):971-984.
27. Wiviott SD, Raz I, Bonaca MP, Mosenzon O, Kato ET, Cahn A, et al. Dapagliflozin and Cardiovascular Outcomes in Type 2 Diabetes. N Engl J Med. 2019;380(4):347-57.
28. Rodbard HW, Bode BW, Harris SB, Rose L, Lehmann L, Jarlov H, et al. Safety and efficacy of insulin degludec/liraglutide (IDegLira) added to sulphonylurea alone or to sulphonylurea and metformin in insulin-naïve people with Type 2 diabetes: the DUAL IV trial. Diabet Med. 2017;34(2):189-96.

29. Bergenstal RM, Wysham C, Macconell L, Malloy J, Walsh B, Yan P, et al. Efficacy and safety of exenatide once weekly versus sitagliptin or pioglitazone as an adjunct to metformin for treatment of type 2 diabetes (DURATION-2): a randomised trial. Lancet. 2010;376(9739):431-9.
30. Russell-Jones D, Cuddihy RM, Hanefeld M, Kumar A, González JG, Chan M, et al. Efficacy and safety of exenatide once weekly versus metformin, pioglitazone, and sitagliptin used as monotherapy in drug-naive patients with type 2 diabetes (DURATION-4): a 26-week double-blind study. Diabetes Care. 2012;35(2):252-8.
31. Guja C, Frías JP, Somogyi A, Jabbour S, Wang H, Hardy E, et al. Effect of exenatide QW or placebo, both added to titrated insulin glargine, in uncontrolled type 2 diabetes: The DURATION-7 randomized study. Diabetes Obes Metab. 2018;20(7):1602-14.
32. Frías JP, Guja C, Hardy E, Ahmed A, Dong F, Öhman P, et al. Exenatide once weekly plus dapagliflozin once daily versus exenatide or dapagliflozin alone in patients with type 2 diabetes inadequately controlled with metformin monotherapy (DURATION-8): a 28 week, multicentre, double-blind, phase 3, randomised controlled trial. Lancet Diabetes Endocrinol. 2016;4(12):1004-16.
33. DeFronzo RA, Hissa MN, Garber AJ, Luiz Gross J, Yuyan Duan R, Ravichandran S, et al. The efficacy and safety of saxagliptin when added to metformin therapy in patients with inadequately controlled type 2 diabetes with metformin alone. Diabetes Care. 2009;32(9):1649-55.
34. Del Prato S, Camisasca R, Wilson C, Fleck P. Durability of the efficacy and safety of alogliptin compared with glipizide in type 2 diabetes mellitus: a 2-year study. Diabetes Obes Metab. 2014;16(12):1239-46.
35. Dormandy JA, Charbonnel B, Eckland DJ, Erdmann E, Massi-Benedetti M, Moules IK, et al. Secondary prevention of macrovascular events in patients with type 2 diabetes in the PROactive Study (PROspective pioglitAzone Clinical Trial In macroVascular Events): a randomised controlled trial. Lancet. 2005;366(9493):1279-89.
36. Pfeffer MA, Claggett B, Diaz R, Dickstein K, Gerstein HC, Køber LV, et al. Lixisenatide in Patients with Type 2 Diabetes and Acute Coronary Syndrome. N Engl J Med. 2015;373(23):2247-57.
37. Zinman B, Wanner C, Lachin JM, Fitchett D, Bluhmki E, Hantel S, et al. Empagliflozin, Cardiovascular Outcomes, and Mortality in Type 2 Diabetes. N Engl J Med. 2015;373(22):2117-28.
38. White WB, Cannon CP, Heller SR, Nissen SE, Bergenstal RM, Bakris GL, et al. Alogliptin after acute coronary syndrome in patients with type 2 diabetes. N Engl J Med. 2013;369(14):1327-35.
39. Holman RR, Bethel MA, Mentz RJ, Thompson VP, Lokhnygina Y, Buse JB, et al. Effects of Once-Weekly Exenatide on Cardiovascular Outcomes in Type 2 Diabetes. N Engl J Med. 2017;377(13):1228-39.
40. Ferdinand KC, White WB, Calhoun DA, Lonn EM, Sager PT, Brunelle R, et al. Effects of the once-weekly glucagon-like peptide-1 receptor agonist dulaglutide on ambulatory blood pressure and heart rate in patients with type 2 diabetes mellitus. Hypertension. 2014;64(4):731-7.
41. Ferrannini E, Fonseca V, Zinman B, Matthews D, Ahrén B, Byiers S, et al. Fifty-two-week efficacy and safety of vildagliptin vs. glimepiride in patients with type 2 diabetes mellitus inadequately controlled on metformin monotherapy. Diabetes Obes Metab. 2009;11(2):157-66.
42. Bolli GB, Munteanu M, Dotsenko S, Niemoeller E, Boka G, Wu Y, et al. Efficacy and safety of lixisenatide once daily vs. placebo in people with Type 2 diabetes insufficiently controlled on metformin (GetGoal-F1). Diabet Med. 2014;31(2):176-84.
43. Gallwitz B, Rosenstock J, Rauch T, Bhattacharya S, Patel S, von Eynatten M, et al. 2-year efficacy and safety of linagliptin compared with glimepiride in patients with type 2 diabetes inadequately controlled on metformin: a randomised, double-blind, non-inferiority trial. Lancet. 2012;380(9840):475-83.

44. Gantz I, Chen M, Suryawanshi S, Ntabadde C, Shah S, O'Neill EA, et al. A randomized, placebo-controlled study of the cardiovascular safety of the once-weekly DPP-4 inhibitor omarigliptin in patients with type 2 diabetes mellitus. Cardiovasc Diabetol. 2017;16(1):112.
45. Riddle MC, Forst T, Aronson R, Sauque-Reyna L, Souhami E, Silvestre L, et al. Adding once-daily lixisenatide for type 2 diabetes inadequately controlled with newly initiated and continuously titrated basal insulin glargine: a 24-week, randomized, placebo-controlled study (GetGoal-Duo 1). Diabetes Care. 2013;36(9):2497-503.
46. Riddle MC, Aronson R, Home P, Marre M, Niemoeller E, Miossec P, et al. Adding once-daily lixisenatide for type 2 diabetes inadequately controlled by established basal insulin: a 24-week, randomized, placebo-controlled comparison (GetGoal-L). Diabetes Care. 2013;36(9):2489-96.
47. Seino Y, Ikeda Y, Niemoeller E, Watanabe D, Takagi H, Yabe D, et al. Efficacy and Safety of Lixisenatide in Japanese Patients with Type 2 Diabetes Insufficiently Controlled with Basal Insulin±Sulfonylurea: A Subanalysis of the GetGoal-L-Asia Study. Horm Metab Res. 2015;47(12):895-900.
48. Yu Pan C, Han P, Liu X, Yan S, Feng P, Zhou Z, et al. Lixisenatide treatment improves glycaemic control in Asian patients with type 2 diabetes mellitus inadequately controlled on metformin with or without sulfonylurea: a randomized, double-blind, placebo-controlled, 24-week trial (GetGoal-M-Asia). Diabetes Metab Res Rev. 2014;30(8):726-35.
49. Meneilly GS, Roy-Duval C, Alawi H, Dailey G, Bellido D, Trescoli C, et al. Lixisenatide Therapy in Older Patients With Type 2 Diabetes Inadequately Controlled on Their Current Antidiabetic Treatment: The GetGoal-O Randomized Trial. Diabetes Care. 2017;40(4):485-93.
50. Rosenstock J, Hanefeld M, Shamanna P, Min KW, Boka G, Miossec P, et al. Beneficial effects of once-daily lixisenatide on overall and postprandial glycemic levels without significant excess of hypoglycemia in type 2 diabetes inadequately controlled on a sulfonylurea with or without metformin (GetGoal-S). J Diabetes Complications. 2014;28(3):386-92.
51. Giles TD, Miller AB, Elkayam U, Bhattacharya M, Perez A. Pioglitazone and heart failure: results from a controlled study in patients with type 2 diabetes mellitus and systolic dysfunction. J Card Fail. 2008;14(6):445-52.
52. Giles TD, Elkayam U, Bhattacharya M, Perez A, Miller AB. Comparison of pioglitazone vs glyburide in early heart failure: insights from a randomized controlled study of patients with type 2 diabetes and mild cardiac disease. Congest Heart Fail. 2010;16(3):111-7.
53. Göke B, Hershon K, Kerr D, Calle Pascual A, Schweizer A, Foley J, et al. Efficacy and safety of vildagliptin monotherapy during 2-year treatment of drug-naïve patients with type 2 diabetes: comparison with metformin. Horm Metab Res. 2008;40(12):892-5.
54. Göke B, Gallwitz B, Eriksson JG, Hellqvist Å, Gause-Nilsson I. Saxagliptin vs. glipizide as add-on therapy in patients with type 2 diabetes mellitus inadequately controlled on metformin alone: long-term (52-week) extension of a 52-week randomised controlled trial. Int J Clin Pract. 2013;67(4):307-16.
55. Hernandez AF, Green JB, Janmohamed S, D'Agostino RB, Granger CB, Jones NP, et al. Albiglutide and cardiovascular outcomes in patients with type 2 diabetes and cardiovascular disease (Harmony Outcomes): a double-blind, randomised placebo-controlled trial. Lancet. 2018;392(10157):1519-29.
56. Reusch J, Stewart MW, Perkins CM, Cirkel DT, Ye J, Perry CR, et al. Efficacy and safety of once-weekly glucagon-like peptide 1 receptor agonist albiglutide (HARMONY 1 trial): 52-week primary endpoint results from a randomized, double-blind, placebo-controlled trial in patients with type 2 diabetes mellitus not controlled on pioglitazone, with or without metformin. Diabetes Obes Metab. 2014;16(12):1257-64.

57. Ahrén B, Johnson SL, Stewart M, Cirkel DT, Yang F, Perry C, et al. HARMONY 3: 104-week randomized, double-blind, placebo- and active-controlled trial assessing the efficacy and safety of albiglutide compared with placebo, sitagliptin, and glimepiride in patients with type 2 diabetes taking metformin. Diabetes Care. 2014;37(8):2141-8.
58. Home PD, Shamanna P, Stewart M, Yang F, Miller M, Perry C, et al. Efficacy and tolerability of albiglutide versus placebo or pioglitazone over 1 year in people with type 2 diabetes currently taking metformin and glimepiride: HARMONY 5. Diabetes Obes Metab. 2015;17(2):179-87.
59. Leiter LA, Carr MC, Stewart M, Jones-Leone A, Scott R, Yang F, et al. Efficacy and safety of the once-weekly GLP-1 receptor agonist albiglutide versus sitagliptin in patients with type 2 diabetes and renal impairment: a randomized phase III study. Diabetes Care. 2014;37(10):2723-30.
60. Haering HU, Merker L, Christiansen AV, Roux F, Salsali A, Kim G, et al. Empagliflozin as add-on to metformin plus sulphonylurea in patients with type 2 diabetes. Diabetes Res Clin Pract. 2015;110(1):82-90.
61. Handelsman Y, Lauring B, Gantz I, Iredale C, O'Neill EA, Wei Z, et al. A randomized, double-blind, non-inferiority trial evaluating the efficacy and safety of omarigliptin, a once-weekly DPP-4 inhibitor, or glimepiride in patients with type 2 diabetes inadequately controlled on metformin monotherapy. Curr Med Res Opin. 2017;33(10):1861-8.
62. Henry RR, Staels B, Fonseca VA, Chou MZ, Teng R, Golm GT, et al. Efficacy and safety of initial combination treatment with sitagliptin and pioglitazone--a factorial study. Diabetes Obes Metab. 2014;16(3):223-30.
63. Hollander PL, Li J, Frederich R, Allen E, Chen R, Investigators C. Safety and efficacy of saxagliptin added to thiazolidinedione over 76 weeks in patients with type 2 diabetes mellitus. Diab Vasc Dis Res. 2011;8(2):125-35.
64. Hollander P, Hill J, Johnson J, Wei Jiang Z, Golm G, Huyck S, et al. Results of VERTIS SU extension study: safety and efficacy of ertugliflozin treatment over 104 weeks compared to glimepiride in patients with type 2 diabetes mellitus inadequately controlled on metformin. Curr Med Res Opin. 2019;35(8):1335-43.
65. Hong J, Zhang Y, Lai S, Lv A, Su Q, Dong Y, et al. Effects of metformin versus glipizide on cardiovascular outcomes in patients with type 2 diabetes and coronary artery disease. Diabetes Care. 2013;36(5):1304-11.
66. Buse JB, Vilsbøll T, Thurman J, Blevins TC, Langbakke IH, Bøttcher SG, et al. Contribution of liraglutide in the fixed-ratio combination of insulin degludec and liraglutide (IDegLira). Diabetes Care. 2014;37(11):2926-33.
67. Jain R, Osei K, Kupfer S, Perez AT, Zhang J. Long-term safety of pioglitazone versus glyburide in patients with recently diagnosed type 2 diabetes mellitus. Pharmacotherapy. 2006;26(10):1388-95.
68. Ji L, Dong X, Li Y, et al. Efficacy and safety of once-weekly semaglutide versus once-daily sitagliptin as add-on to metformin in patients with type 2 diabetes in SUSTAIN China: A 30-week, double-blind, phase 3a, randomized trial. Diabetes Obes Metab. 2021;23(2):404-414.
69. Kawamori R, Haneda M, Suzaki K, Cheng G, Shiki K, Miyamoto Y, et al. Empagliflozin as add-on to linagliptin in a fixed-dose combination in Japanese patients with type 2 diabetes: Glycaemic efficacy and safety profile in a 52-week, randomized, placebo-controlled trial. Diabetes Obes Metab. 2018;20(9):2200-9.
70. Kohan DE, Fioretto P, Tang W, List JF. Long-term study of patients with type 2 diabetes and moderate renal impairment shows that dapagliflozin reduces weight and blood pressure but does not improve glycemic control. Kidney Int. 2014;85(4):962-71.

71. Kovacs CS, Seshiah V, Merker L, Christiansen AV, Roux F, Salsali A, et al. Empagliflozin as Add-on Therapy to Pioglitazone With or Without Metformin in Patients With Type 2 Diabetes Mellitus. Clin Ther. 2015;37(8):1773-88.e1.
72. Marso SP, Daniels GH, Brown-Frandsen K, Kristensen P, Mann JF, Nauck MA, et al. Liraglutide and Cardiovascular Outcomes in Type 2 Diabetes. N Engl J Med. 2016;375(4):311-22.
73. Davies MJ, Bain SC, Atkin SL, Rossing P, Scott D, Shamkhalova MS, et al. Efficacy and Safety of Liraglutide Versus Placebo as Add-on to Glucose-Lowering Therapy in Patients With Type 2 Diabetes and Moderate Renal Impairment (LIRA-RENAL): A Randomized Clinical Trial. Diabetes Care. 2016;39(2):222-30.
74. Bailey TS, Takács R, Tinahones FJ, Rao PV, Tsoukas GM, Thomsen AB, et al. Efficacy and safety of switching from sitagliptin to liraglutide in subjects with type 2 diabetes (LIRA-SWITCH): a randomized, double-blind, double-dummy, active-controlled 26-week trial. Diabetes Obes Metab. 2016;18(12):1191-8.
75. Laakso M, Rosenstock J, Groop PH, Barnett AH, Gallwitz B, Hehnke U, et al. Treatment with the dipeptidyl peptidase-4 inhibitor linagliptin or placebo followed by glimepiride in patients with type 2 diabetes with moderate to severe renal impairment: a 52-week, randomized, double-blind clinical trial. Diabetes Care. 2015;38(2):e15-7.
76. Leiter LA, Cefalu WT, de Bruin TW, Gause-Nilsson I, Sugg J, Parikh SJ. Dapagliflozin added to usual care in individuals with type 2 diabetes mellitus with preexisting cardiovascular disease: a 24-week, multicenter, randomized, double-blind, placebo-controlled study with a 28-week extension. J Am Geriatr Soc. 2014;62(7):1252-62.
77. Leiter LA, Yoon KH, Arias P, Langslet G, Xie J, Balis DA, et al. Canagliflozin provides durable glycemic improvements and body weight reduction over 104 weeks versus glimepiride in patients with type 2 diabetes on metformin: a randomized, double-blind, phase 3 study. Diabetes Care. 2015;38(3):355-64.
78. Lewin A, DeFronzo RA, Patel S, Liu D, Kaste R, Woerle HJ, et al. Initial combination of empagliflozin and linagliptin in subjects with type 2 diabetes. Diabetes Care. 2015;38(3):394-402.
79. Mathieu C, Herrera Marmolejo M, González González JG, Hansen L, Chen H, Johnsson E, et al. Efficacy and safety of triple therapy with dapagliflozin add-on to saxagliptin plus metformin over 52 weeks in patients with type 2 diabetes. Diabetes Obes Metab. 2016;18(11):1134-7.
80. Matthews DR, Dejager S, Ahren B, Fonseca V, Ferrannini E, Couturier A, et al. Vildagliptin add-on to metformin produces similar efficacy and reduced hypoglycaemic risk compared with glimepiride, with no weight gain: results from a 2-year study. Diabetes Obes Metab. 2010;12(9):780-9.
81. Matthews DR, Paldánius PM, Proot P, Chiang Y, Stumvoll M, Del Prato S, et al. Glycaemic durability of an early combination therapy with vildagliptin and metformin versus sequential metformin monotherapy in newly diagnosed type 2 diabetes (VERIFY): a 5-year, multicentre, randomised, double-blind trial. Lancet. 2019;394(10208):1519-29.
82. Mazzone T, Meyer PM, Feinstein SB, Davidson MH, Kondos GT, D'Agostino RB, et al. Effect of pioglitazone compared with glimepiride on carotid intima-media thickness in type 2 diabetes: a randomized trial. JAMA. 2006;296(21):2572-81.
83. Merker L, Häring HU, Christiansen AV, Roux F, Salsali A, Kim G, et al. Empagliflozin as add-on to metformin in people with Type 2 diabetes. Diabet Med. 2015;32(12):1555-67.
84. Moses RG, Round E, Shentu Y, Golm GT, O'neill EA, Gantz I, et al. A randomized clinical trial evaluating the safety and efficacy of sitagliptin added to the combination of sulfonylurea and metformin in patients with type 2 diabetes mellitus and inadequate glycemic control. J Diabetes. 2016;8(5):701-11.

85. Müller-Wieland D, Kellerer M, Cypryk K, Skripova D, Rohwedder K, Johnsson E, et al. Efficacy and safety of dapagliflozin or dapagliflozin plus saxagliptin versus glimepiride as add-on to metformin in patients with type 2 diabetes. Diabetes Obes Metab. 2018;20(11):2598-607.
86. Nauck MA, di Domenico M, Patel S, Kobe M, Toorawa R, Woerle HJ. Linagliptin and pioglitazone combination therapy versus monotherapy with linagliptin or pioglitazone: A randomised, double-blind, parallel-group, multinational clinical trial. Diab Vasc Dis Res. 2016;13(4):286-98.
87. Nissen SE, Nicholls SJ, Wolski K, Nesto R, Kupfer S, Perez A, et al. Comparison of pioglitazone vs glimepiride on progression of coronary atherosclerosis in patients with type 2 diabetes: the PERISCOPE randomized controlled trial. JAMA. 2008;299(13):1561-73.
88. Olansky L, Reasner C, Seck TL, Williams-Herman DE, Chen M, Terranella L, et al. A treatment strategy implementing combination therapy with sitagliptin and metformin results in superior glycaemic control versus metformin monotherapy due to a low rate of addition of antihyperglycaemic agents. Diabetes Obes Metab. 2011;13(9):841-9.
89. Rosenstock J, Allison D, Birkenfeld AL, Blicher TM, Deenadayalan S, Jacobsen JB, et al. Effect of Additional Oral Semaglutide vs Sitagliptin on Glycated Hemoglobin in Adults With Type 2 Diabetes Uncontrolled With Metformin Alone or With Sulfonylurea: The PIONEER 3 Randomized Clinical Trial. JAMA. 2019;321(15):1466-80.
90. Husain M, Birkenfeld AL, Donsmark M, Dungan K, Eliaschewitz FG, Franco DR, et al. Oral Semaglutide and Cardiovascular Outcomes in Patients with Type 2 Diabetes. N Engl J Med. 2019;381(9):841-51.
91. Zinman B, Aroda VR, Buse JB, Cariou B, Harris SB, Hoff ST, et al. Efficacy, Safety, and Tolerability of Oral Semaglutide Versus Placebo Added to Insulin With or Without Metformin in Patients With Type 2 Diabetes: The PIONEER 8 Trial. Diabetes Care. 2019;42(12):2262-71.
92. Perez A, Jacks R, Arora V, Spanheimer R. Effects of pioglitazone and metformin fixed-dose combination therapy on cardiovascular risk markers of inflammation and lipid profile compared with pioglitazone and metformin monotherapy in patients with type 2 diabetes. J Clin Hypertens (Greenwich). 2010;12(12):973-82.
93. Perkovic V, Jardine MJ, Neal B, Bompoint S, Heerspink HJL, Charytan DM, et al. Canagliflozin and Renal Outcomes in Type 2 Diabetes and Nephropathy. N Engl J Med. 2019;380(24):2295-306.
94. Pfützner A, Paz-Pacheco E, Allen E, Frederich R, Chen R, Investigators C. Initial combination therapy with saxagliptin and metformin provides sustained glycaemic control and is well tolerated for up to 76 weeks. Diabetes Obes Metab. 2011;13(6):567-76.
95. Pinget M, Goldenberg R, Niemoeller E, Muehlen-Bartmer I, Guo H, Aronson R. Efficacy and safety of lixisenatide once daily versus placebo in type 2 diabetes insufficiently controlled on pioglitazone (GetGoal-P). Diabetes Obes Metab. 2013;15(11):1000-7.
96. Pratley RE, Eldor R, Raji A, Golm G, Huyck SB, Qiu Y, et al. Ertugliflozin plus sitagliptin versus either individual agent over 52 weeks in patients with type 2 diabetes mellitus inadequately controlled with metformin: The VERTIS FACTORIAL randomized trial. Diabetes Obes Metab. 2018;20(5):1111-20.
97. Gerstein HC, Colhoun HM, Dagenais GR, Diaz R, Lakshmanan M, Pais P, et al. Dulaglutide and cardiovascular outcomes in type 2 diabetes (REWIND): a double-blind, randomised placebo-controlled trial. Lancet. 2019;394(10193):121-30.
98. Ridderstråle M, Rosenstock J, Andersen KR, Woerle HJ, Salsali A, investigators E-RHH-St. Empagliflozin compared with glimepiride in metformin-treated patients with type 2 diabetes: 208-week data from a masked randomized controlled trial. Diabetes Obes Metab. 2018;20(12):2768-77.

99. Roden M, Merker L, Christiansen AV, Roux F, Salsali A, Kim G, et al. Safety, tolerability and effects on cardiometabolic risk factors of empagliflozin monotherapy in drug-naïve patients with type 2 diabetes: a double-blind extension of a Phase III randomized controlled trial. Cardiovasc Diabetol. 2015;14:154.
100. Rosenstock J, Wilson C, Fleck P. Alogliptin versus glipizide monotherapy in elderly type 2 diabetes mellitus patients with mild hyperglycaemia: a prospective, double-blind, randomized, 1-year study. Diabetes Obes Metab. 2013;15(10):906-14.
101. Rosenstock J, Jelaska A, Frappin G, Salsali A, Kim G, Woerle HJ, et al. Improved glucose control with weight loss, lower insulin doses, and no increased hypoglycemia with empagliflozin added to titrated multiple daily injections of insulin in obese inadequately controlled type 2 diabetes. Diabetes Care. 2014;37(7):1815-23.
102. Rosenstock J, Jelaska A, Zeller C, Kim G, Broedl UC, Woerle HJ, et al. Impact of empagliflozin added on to basal insulin in type 2 diabetes inadequately controlled on basal insulin: a 78-week randomized, double-blind, placebo-controlled trial. Diabetes Obes Metab. 2015;17(10):936-48.
103. Rosenstock J, Perl S, Johnsson E, García-Sánchez R, Jacob S. Triple therapy with low-dose dapagliflozin plus saxagliptin versus dual therapy with each monocomponent, all added to metformin, in uncontrolled type 2 diabetes. Diabetes Obes Metab. 2019;21(9):2152-62.
104. Bhatt DL, Szarek M, Pitt B, et al. Sotagliflozin in Patients with Diabetes and Chronic Kidney Disease. N Engl J Med. 2021;384(2):129-139.
105. Scirica BM, Bhatt DL, Braunwald E, Steg PG, Davidson J, Hirshberg B, et al. Saxagliptin and cardiovascular outcomes in patients with type 2 diabetes mellitus. N Engl J Med. 2013;369(14):1317-26.
106. Davies MJ, Bergenstal R, Bode B, et al. Efficacy of Liraglutide for Weight Loss Among Patients With Type 2 Diabetes: The SCALE Diabetes Randomized Clinical Trial [published correction appears in JAMA. 2016 Jan 5;315(1):90]. JAMA. 2015;314(7):687-699.
107. Sorli C, Harashima SI, Tsoukas GM, Unger J, Karsbøl JD, Hansen T, et al. Efficacy and safety of once-weekly semaglutide monotherapy versus placebo in patients with type 2 diabetes (SUSTAIN 1): a double-blind, randomised, placebo-controlled, parallel-group, multinational, multicentre phase 3a trial. Lancet Diabetes Endocrinol. 2017;5(4):251-60.
108. Marso SP, Bain SC, Consoli A, Eliaschewitz FG, Jódar E, Leiter LA, et al. Semaglutide and Cardiovascular Outcomes in Patients with Type 2 Diabetes. N Engl J Med. 2016;375(19):1834-44.
109. Lingvay I, Catarig AM, Frias JP, Kumar H, Lausvig NL, le Roux CW, et al. Efficacy and safety of once-weekly semaglutide versus daily canagliflozin as add-on to metformin in patients with type 2 diabetes (SUSTAIN 8): a double-blind, phase 3b, randomised controlled trial. Lancet Diabetes Endocrinol. 2019;7(11):834-44.
110. Schernthaner G, Gross JL, Rosenstock J, Guarisco M, Fu M, Yee J, et al. Canagliflozin compared with sitagliptin for patients with type 2 diabetes who do not have adequate glycemic control with metformin plus sulfonylurea: a 52-week randomized trial. Diabetes Care. 2013;36(9):2508-15.
111. Schernthaner G, Durán-Garcia S, Hanefeld M, Langslet G, Niskanen L, Östgren CJ, et al. Efficacy and tolerability of saxagliptin compared with glimepiride in elderly patients with type 2 diabetes: a randomized, controlled study (GENERATION). Diabetes Obes Metab. 2015;17(7):630-8.
112. Schweizer A, Dejager S, Bosi E. Comparison of vildagliptin and metformin monotherapy in elderly patients with type 2 diabetes: a 24-week, double-blind, randomized trial. Diabetes Obes Metab. 2009;11(8):804-12.

113. Scott R, Morgan J, Zimmer Z, Lam RLH, O'Neill EA, Kaufman KD, et al. A randomized clinical trial of the efficacy and safety of sitagliptin compared with dapagliflozin in patients with type 2 diabetes mellitus and mild renal insufficiency: The CompoSIT-R study. Diabetes Obes Metab. 2018;20(12):2876-84.
114. Seck T, Nauck M, Sheng D, Sunga S, Davies MJ, Stein PP, et al. Safety and efficacy of treatment with sitagliptin or glipizide in patients with type 2 diabetes inadequately controlled on metformin: a 2-year study. Int J Clin Pract. 2010;64(5):562-76.
115. Seino Y, Rasmussen MF, Nishida T, Kaku K. Efficacy and safety of the once-daily human GLP-1 analogue, liraglutide, vs glibenclamide monotherapy in Japanese patients with type 2 diabetes. Curr Med Res Opin. 2010;26(5):1013-22.
116. Seino Y, Kaneko S, Fukuda S, Osonoi T, Shiraiwa T, Nishijima K, et al. Combination therapy with liraglutide and insulin in Japanese patients with type 2 diabetes: A 36-week, randomized, double-blind, parallel-group trial. J Diabetes Investig. 2016;7(4):565-73.
117. Bhatt DL, Szarek M, Steg PG, et al. Sotagliflozin in Patients with Diabetes and Recent Worsening Heart Failure. N Engl J Med. 2021;384(2):117-128.
118. Strojek K, Yoon KH, Hruba V, Sugg J, Langkilde AM, Parikh S. Dapagliflozin added to glimepiride in patients with type 2 diabetes mellitus sustains glycemic control and weight loss over 48 weeks: a randomized, double-blind, parallel-group, placebo-controlled trial. Diabetes Ther. 2014;5(1):267-83.
119. Green JB, Bethel MA, Armstrong PW, Buse JB, Engel SS, Garg J, et al. Effect of Sitagliptin on Cardiovascular Outcomes in Type 2 Diabetes. N Engl J Med. 2015;373(3):232-42.
120. Tolman KG, Freston JW, Kupfer S, Perez A. Liver safety in patients with type 2 diabetes treated with pioglitazone: results from a 3-year, randomized, comparator-controlled study in the US. Drug Saf. 2009;32(9):787-800.
121. Duckworth W, Abraira C, Moritz T, Reda D, Emanuele N, Reaven PD, et al. Glucose control and vascular complications in veterans with type 2 diabetes. N Engl J Med. 2009;360(2):129-39.
122. Wainstein J, Katz L, Engel SS, Xu L, Golm GT, Hussain S, et al. Initial therapy with the fixed-dose combination of sitagliptin and metformin results in greater improvement in glycaemic control compared with pioglitazone monotherapy in patients with type 2 diabetes. Diabetes Obes Metab. 2012;14(5):409-18.
123. Wilding JP, Woo V, Soler NG, Pahor A, Sugg J, Rohwedder K, et al. Long-term efficacy of dapagliflozin in patients with type 2 diabetes mellitus receiving high doses of insulin: a randomized trial. Ann Intern Med. 2012;156(6):405-15.
124. Wilding JP, Woo V, Soler NG, Pahor A, Sugg J, Rohwedder K, et al. [Long-term efficacy of dapagliflozin in patients with type 2 diabetes mellitus receiving high doses of insulin]. Dtsch Med Wochenschr. 2013;138 Suppl 1:S27-38.
125. Yale JF, Bakris G, Cariou B, Nieto J, David-Neto E, Yue D, et al. Efficacy and safety of canagliflozin over 52 weeks in patients with type 2 diabetes mellitus and chronic kidney disease. Diabetes Obes Metab. 2014;16(10):1016-27.
126. Yang W, Ma J, Li Y, Zhou Z, Kim JH, Zhao J, et al. Dapagliflozin as add-on therapy in Asian patients with type 2 diabetes inadequately controlled on insulin with or without oral antihyperglycemic drugs: A randomized controlled trial. J Diabetes. 2018;10(7):589-99.
