# Supplementary material for: Glucose-Lowering and the Risk of Cardiovascular Events With Antidiabetic Therapies: A Systematic Review and Additive-Effects Network Meta-Analysis
Source: Front Cardiovasc Med. 2022 Apr 29;9:876795. doi: 10.3389/fcvm.2022.876795 (PMC9098935; doi:10.3389/fcvm.2022.876795)
Supplement: Supplementary file 2 [file Data_Sheet_1.PDF]

Oral antidiabetic medications (OAD) vs Optimized Standard Therapy (TPO) and Hospitalisation for Heart Failure (HHF) in patients with type 2 diabetes (T2DM): Network Meta-analysis

Riobaldo Cintra, Ana Nogueira, Isabella Bonilha, Luiz Sérgio Carvalho, Beatriz Luchiari, Andrei Sposito

To enable PROSPERO to focus on COVID-19 registrations during the 2020 pandemic, this registration record was automatically published exactly as submitted. The PROSPERO team has not checked eligibility.

Citation

Riobaldo Cintra, Ana Nogueira, Isabella Bonilha, Luiz Sérgio Carvalho, Beatriz Luchiari, Andrei Sposito. Oral antidiabetic medications (OAD) vs Optimized Standard Therapy (TPO) and Hospitalisation for Heart Failure (HHF) in patients with type 2 diabetes (T2DM): Network Meta-analysis. PROSPERO 2020 CRD42020213127 Available from:
https://www.crd.york.ac.uk/prospero/display_record.php?ID=CRD42020213127

Review question

Are Oral Antidiabetic (OAD) different in terms of the incidence of Hospitalisation for Heart Failure (HHF) in patients with type 2 diabetes (T2DM)?

Does the use of DPP4 inhibitors in patients with T2DM change HHF?

Does the use of the DPP4 inhibitors plus SGLT2 inhibitors in patients with T2DM change HHF?

Does the use of the SGLT2 inhibitors plus Metformin in patients with T2DM change HHF?

Does the use of Thiazolidinediones (TZDs) in patients with T2DM change HHF?

Does the use of Thiazolidinediones (TZDs) plus DPP4 inhibitors in patients with T2DM change HHF?

Does the use of Sulphonylurea in patients with T2DM change HHF?

Searches

Inhibitors of sodium–glucose cotransporter 2, dipeptidyl peptidase 4 (DPP-4) inhibitors, Sulphonylureas, thiazolidinedione, type 2 Diabetes, hospitalization for heart failure

Papers publicated untill july/2020

Types of study to be included

Randomized clinical trials phase 3 or 4, involving patients with T2DM, sample ?100 patients per arm and follow-up ?24 weeks

Condition or domain being studied

Type 2 Diabetes, Heart Failure, Hospitalisation for Heart Failure

Participants/population

Type 2 Diabetes Individuals

Intervention(s), exposure(s)

Sodium-glucose cotransporter 2 inhibitors, metformin, sulphonylurea, thiazolidinedione, or dipeptidyl peptidase 4 (DPP-4) inhibitors, or a combination of these drugs

Comparator(s)/control

Optimized Standard Therapy

Main outcome(s)
Hospitalisation for heart failure

* Measures of effect

Outcome reported as hazard ratio, relative risk or mean difference

Additional outcome(s)
Severe hypoglycemia, pancreatitis, thyroid cancer, pancreatic caancer, gastrointestinal symptoms, systemic

allergic reaction, amputation, fracture, urinary tract infection and genital tract infection

* Measures of effect

Outcome reported as hazard ratio, relative risk or mean difference

Data extraction (selection and coding)
Four investigators who will not be involved in any of the selected studies, independently, will abstract data using pre-specified forms and independently assess the accuracy of abstractions and resolve any discrepancies by consensus after discussion with the fifth investigator, the study coordinator. The following data will be extracted from the studies: name of the first author, year of publication, sample size, duration of the intervention, patient characteristics, type of control, medication dose, clinical outcomes and adverse events. In the event of an essay published more than once, the last report will be included.

Risk of bias (quality) assessment
To assess the risk of bias, the Cochrane Collaboration tool will be used to assess different forms of bias within the studies included in our meta-analysis. The quality of the study will be assessed with the “Grading of Recommendations Assessment, Development and Evaluation - GRADE". Recommendations Reviews of Interventions, based on allocation concealment (randomization), blinding (performance and detection bias) and attrition bias. The studies included were also judged in accordance with other similar evidence-based medicine classification criteria (e.g., Delphi’s List).

Strategy for data synthesis
The data extracted from the studies will be inserted in a Microsoft Excel® spreadsheet, where the first analysis will be performed. After the initial adjustments, the data will migrate to the R software. There are several statistical methods for making indirect comparisons that can combine the data from the preliminary studies when they are limited or simply non-existent. The network meta-analysis can be performed under a frequentist or Bayesian structure, with several models proposed in both structures. In the present study, an option was made for the hierarchical Bayesian structure for indirect comparison between treatments. The gemtc package for R was chosen to perform the meta-analysis, as the package provides a comprehensive set of tools and important features, such as its ability to map heterogeneity and inconsistency.

Analysis of subgroups or subsets
We will analyze in parallel the randomized clinical trials in which either arm reached a mean HbA1c at the end of the study of less than 7.0%. In parallel, we will separately analyze studies that included patients without previous cardiovascular disease

Contact details for further information
Riobaldo Cintra
riocintra@gmail.com

Organisational affiliation of the review
Atherosclerosis and Vascular Biology Laboratory, State University of Campinas

Review team members and their organisational affiliations
Mr Riobaldo Cintra. Instituto Hospital de Base do Distrito Federal
Ana Nogueira. Instituto Hospital de Base do Distrito Federal

Isabella Bonilha. Faculdade de Ciências Médicas da UNICAMP
Luiz Sérgio Carvalho. Faculdade de Ciências Médicas da UNICAMP
Beatriz Luchiari. Faculdade de Ciências Médicas da UNICAMP
Andrei Sposito. Faculdade de Ciências Médicas da UNICAMP

Type and method of review
Meta-analysis, Network meta-analysis, Systematic review

Anticipated or actual start date
08 October 2020

Anticipated completion date
10 November 2020

Funding sources/sponsors
Own financing

Grant number(s)

State the funder, grant or award number and the date of award

Not applicable

Conflicts of interest

Language
English

Country
Brazil

Stage of review
Review Ongoing

Subject index terms status
Subject indexing assigned by CRD

Subject index terms
MeSH headings have not been applied to this record

Date of registration in PROSPERO
08 November 2020

Date of first submission
08 October 2020

Stage of review at time of this submission

Stage	Started	Completed
Preliminary searches	No	No
Piloting of the study selection process	Yes	No
Formal screening of search results against eligibility criteria	No	No
Data extraction	No	No
Risk of bias (quality) assessment	No	No
Data analysis	No	No

The record owner confirms that the information they have supplied for this submission is accurate and complete and they understand that deliberate provision of inaccurate information or omission of data may be construed as scientific misconduct.

The record owner confirms that they will update the status of the review when it is completed and will add publication details in due course.

Versions

08 November 2020

PROSPERO
This information has been provided by the named contact for this review. CRD has accepted this information in good faith and registered the review in PROSPERO. The registrant confirms that the information supplied for this submission is accurate and complete. CRD bears no responsibility or liability for the content of this registration record, any associated files or external websites.
